# Restoring the Molecular Clockwork within the Suprachiasmatic Hypothalamus of an Otherwise Clockless Mouse Enables Circadian Phasing and Stabilization of Sleep-Wake Cycles and Reverses Memory Deficits

**DOI:** 10.1523/JNEUROSCI.3141-20.2021

**Published:** 2021-10-13

**Authors:** Elizabeth S. Maywood, Johanna E. Chesham, Raphaelle Winsky-Sommerer, Michael H. Hastings

**Affiliations:** ^1^Division of Neurobiology, Medical Research Council Laboratory of Molecular Biology, Cambridge CB2 0QH, United Kingdom; ^2^Surrey Sleep Research Centre, Faculty of Health and Medical Sciences, University of Surrey, Guildford GU2 7XP, United Kingdom

**Keywords:** circadian, clock, cryptochrome, hippocampus, NREM sleep, sleep homeostasis

## Abstract

The timing and quality of sleep-wake cycles are regulated by interacting circadian and homeostatic mechanisms. Although the suprachiasmatic nucleus (SCN) is the principal clock, circadian clocks are active across the brain and the respective sleep-regulatory roles of SCN and local clocks are unclear. To determine the specific contribution(s) of the SCN, we used virally mediated genetic complementation, expressing Cryptochrome1 (Cry1) to establish circadian molecular competence in the suprachiasmatic hypothalamus of globally clockless, arrhythmic male *Cry1/Cry2*-null mice. Under free-running conditions, the rest/activity behavior of *Cry1/Cry2*-null controls expressing EGFP (SCN^Con^) was arrhythmic, whereas Cry1-complemented mice (SCN^Cry1^) had coherent circadian behavior, comparable to that of Cry1,2-competent wild types (WTs). In SCN^Con^ mice, sleep-wakefulness, assessed by electroencephalography (EEG)/electromyography (EMG), lacked circadian organization. In SCN^Cry1^ mice, however, it matched WTs, with consolidated vigilance states [wake, rapid eye movement sleep (REMS) and non-REMS (NREMS)] and rhythms in NREMS δ power and expression of REMS within total sleep (TS). Wakefulness in SCN^Con^ mice was more fragmented than in WTs, with more wake-NREMS-wake transitions. This disruption was reversed in SCN^Cry1^ mice. Following sleep deprivation (SD), all mice showed a homeostatic increase in NREMS δ power, although the SCN^Con^ mice had reduced NREMS during the inactive (light) phase of recovery. In contrast, the dynamics of homeostatic responses in the SCN^Cry1^ mice were comparable to WTs. Finally, SCN^Con^ mice exhibited poor sleep-dependent memory but this was corrected in SCN^Cry1^mice. In clockless mice, circadian molecular competence focused solely on the SCN rescued the architecture and consolidation of sleep-wake and sleep-dependent memory, highlighting its dominant role in timing sleep.

**SIGNIFICANCE STATEMENT** The circadian timing system regulates sleep-wake cycles. The hypothalamic suprachiasmatic nucleus (SCN) is the principal circadian clock, but the presence of multiple local brain and peripheral clocks mean the respective roles of SCN and other clocks in regulating sleep are unclear. We therefore used virally mediated genetic complementation to restore molecular circadian functions in the suprachiasmatic hypothalamus, focusing on the SCN, in otherwise genetically clockless, arrhythmic mice. This initiated circadian activity-rest cycles, and circadian sleep-wake cycles, circadian patterning to the intensity of non-rapid eye movement sleep (NREMS) and circadian control of REMS as a proportion of total sleep (TS). Consolidation of sleep-wake established normal dynamics of sleep homeostasis and enhanced sleep-dependent memory. Thus, the suprachiasmatic hypothalamus, alone, can direct circadian regulation of sleep-wake.

## Introduction

The timing and quality of sleep are determined by a circadian process that ensures sleep occurs appropriately within the light-dark (LD) cycle and a homeostatic process that tracks sleep need during wakefulness (Borbély and Achermann, 1999; Borbély et al., 2016). Whereas the identity of the homeostat remains unknown, the hypothalamic suprachiasmatic nucleus (SCN), is conventionally thought to mediate circadian control (Saper et al., 2005). At the molecular level, the SCN clock consists of transcriptional/posttranslational feedback loops (TTFL) in which *Period* (*Per*) and *Cryptochrome* (*Cry*) genes are trans-activated by CLOCK and BMAL1 heterodimers (Takahashi, 2017). Following their accumulation over circadian day, the encoded Per and Cry proteins inhibit trans-activation, closing the loop, while their subsequent degradation over circadian night allows the cycle to recommence. This TTFL is entrained to solar time by rods, cones and melanopsin-containing retinal ganglion cells innervating the SCN (Berson et al., 2002; Hattar et al., 2002, 2003). In constant darkness (DD), the TTFL runs to its intrinsic approximately 24 h period. Importantly, the TTFL is active in all tissues, including brain regions that regulate the sleep/wake cycle and memory (Hastings et al., 2018). The question arises, therefore, as to whether circadian control of sleep is mediated uniquely by the SCN, or do local brain and peripheral clocks also contribute? Beyond that, the influence of the circadian system (SCN and/or local clocks) on sleep-wake cycles remains to be established: does it only affect timing, or does it modify its temporal architecture and/or homeostatic responses (Gillette, 2004)?

Loss-of-function ablation has demonstrated the necessity of the SCN for circadian timing of sleep under DD, and its redundancy in global sleep homeostasis (Tobler et al., 1983; Mistlberger, 2005), although increased non rapid-eye-movement sleep (NREMS) in SCN-ablated mice suggests a broader role in sleep regulation (Easton et al., 2004). Loss-of-genetic function approaches have also been used to interrogate circadian sleep control. *Per1/Per2*-null mice have a defective TTFL and arrhythmic sleep-wake patterns under DD, but not LD (Shiromani et al., 2004). Moreover, sleep deprivation (SD) induces time of day-dependent expression of *Per1* and *Per2* in the forebrain (Franken et al., 2007; Curie et al., 2013), but *Per1/Per2*-null mice show normal homeostatic regulation of the daily amounts of waking, NREMS, or REMS (Shiromani et al., 2004). Conversely, CLOCK mutant mice show altered homeostatic regulation in the amount of sleep, with less NREMS in both LD and DD (Naylor et al., 2000). Finally, *Cry1/Cry2*-null mice lack TTFL function and so have no circadian pattern to sleep-wake in DD conditions, but show higher levels of NREMS and electroencephalography (EEG) δ power (Wisor et al., 2002). Further, when sleep-deprived under a LD schedule, *Cry1/Cry2*-null mice show less NREMS recovery than do wild-type (WT) mice, suggesting a requirement for Cry proteins in sleep homeostasis. Altogether, this highlights the disparity in the literature on the precise role(s) of the circadian system in regulating sleep/wake cycles.

Untangling the anatomic (SCN, extra-SCN) and genetic (*Per*, *Cry*, *Clock*) contributions to sleep regulation has therefore been challenging for loss-of-function approaches. Interpretation of results from clock gene mutants is constrained because global mutations compromise both the SCN and local clocks. Furthermore, as transcription factors, their encoded proteins may have non-circadian roles. Equally, SCN ablations disrupt neural circuitry and may compromise non-circadian processes (Mistlberger, 2005). Therefore, we chose a gain-of-function approach. Behavioral arrhythmia in clock mutant mice can be rescued by SCN grafting (Sujino et al., 2003) or by virally mediated genetic complementation targeted toward the SCN (Fuller et al., 2008; Maywood et al., 2018). We therefore tested the hypothesis that a molecularly competent SCN clock would be sufficient to co-ordinate phasing and stabilization of sleep-wake cycles, leading to improved sleep-dependent memory in an otherwise clockless and arrhythmic mouse. To do this we used adeno-associated viral (AAV) vectors to express control EGFP or a Cry1::EGFP fusion targeted at the SCN of *Cry1/Cry2*-null mice, leaving local clocks across the brain and periphery dysfunctional.

## Materials and Methods

### Animals and housing

All experiments were conducted in accordance with the UK Animals (Scientific Procedures) Act of 1986, with local ethical approval (MRC LMB, AWERB). We used four- to six-month-old male WT mice and *Cry1*,*Cry2* double knock-out (CDKO) mice (van der Horst et al., 1999), all on a C57/BL/6J genetic background. There were no significant differences in body weights at the time of surgery (WT = 30.5 ± 1.6 g; CDKO = 28.5 ± 1.1 g, *n* = 5, 18, respectively). Mice were housed individually and their activity patterns were monitored continuously using running-wheels. Food and water were provided *ad libitum*. Mice were entrained to a 12/12 h light/dim red light cycle (LD) for at least 10 d before transfer to a schedule of continuous dim red light (DD) for 14 d for assessment of (ar)rhythmicity (DD1). Following surgery (see below), mice were maintained on a 12L:12D photoschedule for recovery before transfer to a second period of DD (DD2). In all of our studies, Zeitgeber time (ZT)0 denotes the time of lights-on and ZT12 lights-off under LD, whereas circadian time (CT)0 denotes the start of subjective day, and CT12 denotes the start of subjective night in DD, as evidenced by activity onset.

### Stereotaxic injection of AAV vector and implantation of EEG/electromyography (EMG) transmitters

Mice were anaesthetized using isoflurane (induction 2–4%; maintenance 1%) with body temperature thermostatically controlled using a heating pad. Rimadyl was used for postoperative analgesia. Under aseptic conditions, the animals received bilateral stereotaxic injections (0.3 µl/site) into the SCN (±0.25 mm medio-lateral to bregma, 5.5 mm deep to dural surface) of a pan-cellular AAV-1 vector encoding pCry1-Cry1::EGFP (3.26x10^12^cg/ml) for circadian expression of Cry1::EGFP fusion, driven by its minimal promoter (SCN^Cry1^, *n* = 9), or pCry1-EGFP control (4.8x10^12^cg/ml; SCN^Con^, *n* = 9; Maywood et al., 2013; Edwards et al., 2016). At the same time, a telemetric transmitter (TL11M2-F20-EET, Data Sciences International) connected to electrodes for continuous EEG and EMG recordings was implanted subcutaneously. Two screws were implanted above the dura (+1.5 mm anterior to bregma and +1.7 mm lateral to bregma, the second +1.0 mm anterior and +1.7 mm lateral to lambda, i.e., over the right hemisphere) around which the electrodes for measuring the EEG were placed and secured using dental cement (RelyX Unicem 2 automix; Henry Schein Medical Animal Health). The two EMG leads were inserted into the trapezius muscle ∼5 mm apart and sutured in place (Hasan et al., 2011; Lang et al., 2011). All mice were allowed 10–14 d of recovery following surgery. To confirm AAV targeting of the SCN, at the conclusion of the study, mice were culled and the brains dissected, fixed in 4% paraformaldehyde in phosphate buffer, cryopreserved overnight in 20% sucrose in PBS and then sectioned (40 µm) on a freezing sledge microtome (Bright Instruments). Confocal microscopy (Zeiss 780 inverted confocal system) of the native EGFP signal in control SCN^Con^ and SCN^Cry1^ groups was used to identify successful targeting of the SCN. Brain sections were mounted onto slides and coverslipped using Vectashield Hardset mounting medium with DAPI (Vector Labs, RRID:AB_2336788). Cell counts in SCN sections were assessed by the ratio of EGFP-positive cells to DAPI-positive cells using ImageJ. Because of inefficient targeting, two of the nine animals were removed from further analysis in the SCN^Cry1^ group and one from the SCN^Con^ group.

### EEG/EMG recordings and determination of vigilance states and spectral analysis

**Figure 1.**
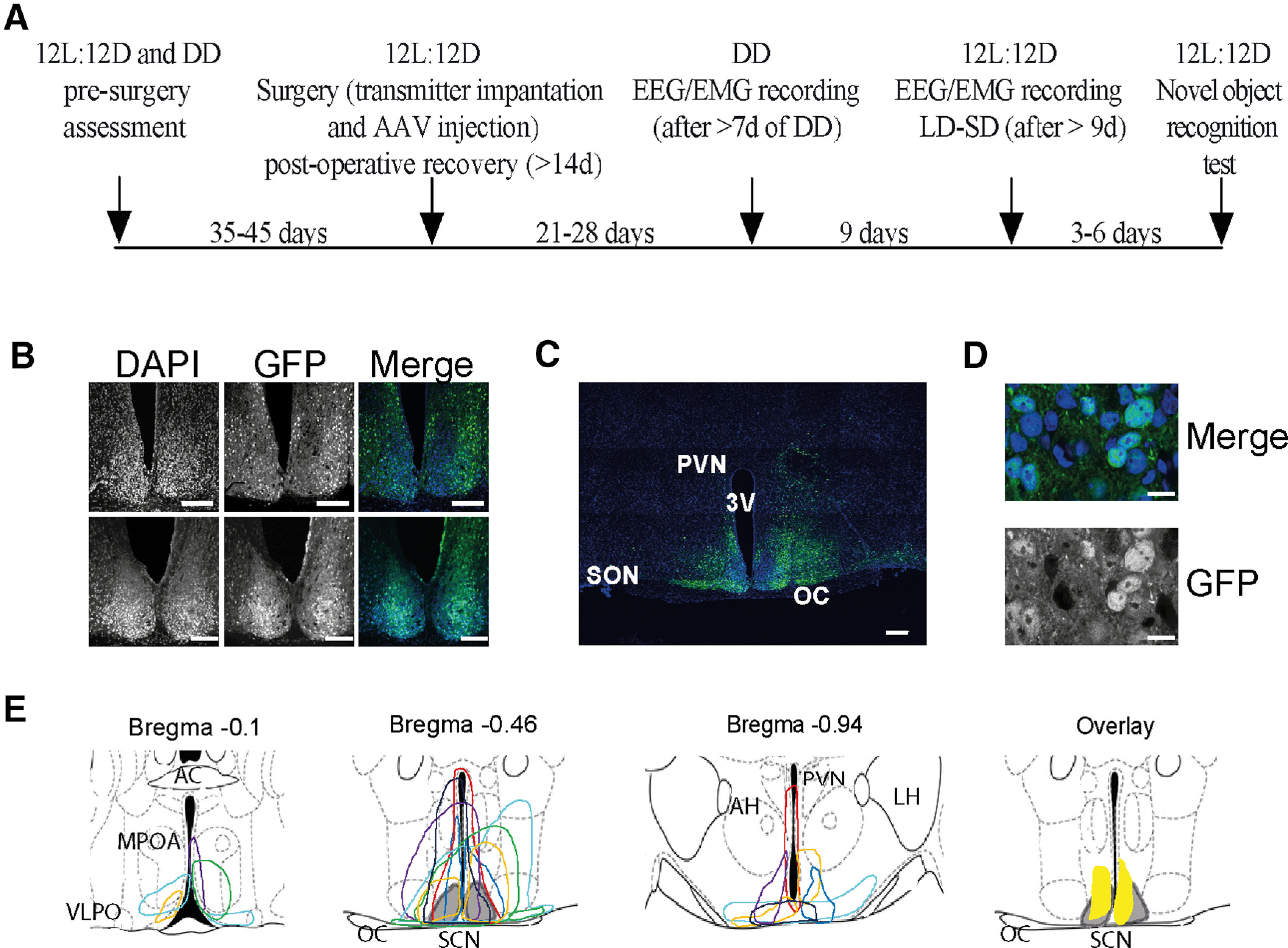
Histologic confirmation of the expression of pCry1-Cry1-EGFP in the suprachiasmatic hypothalamus of Cry1,2-null mice used in the sleep studies. ***A***, Timeline showing the order of experimental procedures and interventions. The number of days between each stage is listed. ***B***, Fluorescence confocal images from the brains of two mice injected with an AAV (pCry1-CRY1::EGFP) to restore CRY1 into the SCN (SCN^Cry1^); 20×; scale bar: 150 μm. ***C***, Tiled (4 × 4) fluorescence confocal image from the same animal as in the upper panel of ***B***; 20×; scale bar: 150 μm. ***D***, High-power fluorescence confocal image from the same animal as in the upper panel of ***B***, showing the nuclear localization of the GFP signal; 63×; scale bar: 10 μm. ***E***, Color-coded depiction of the area of AAV-pCry1-CRY1::EGFP expression within the SCN and surrounding hypothalamus represented on coronal schematics modified from a mouse brain atlas (Paxinos and Franklin, 2001). Each color represents a single mouse used in the sleep studies. The gray shaded area represents the SCN. The yellow shaded area in the overlay plot shows the targeted area common to all mice. AC, anterior commissure; AH, anterior hypothalamus; MPOA, medial preoptic area; OC, optic chiasm; OT, optic tract; PVN, paraventricular nucleus; VLPO, ventrolateral preoptic nucleus.

Transmitters were activated on the day before data collection and EEG/EMG were recorded continuously from the freely moving animals in both DD (3 d; >21 d postsurgery; Fig. 1*A*) and LD (2 d; >30–37 d postsurgery) using Data Sciences International hardware and Dataquest ART v2.3 Gold software. Vigilance states for consecutive 4-s epochs were classified by visual inspection according to standard criteria: wakefulness (high and variable EMG signal, low-amplitude EEG signal), NREMS (high EEG amplitude dominated by slow waves, low EMG), and REMS (low EEG amplitude, theta oscillations and muscle atonia). Vigilance states were analyzed offline using Neuroscore Software (v.2.1 Data Sciences International) with the EEG and EMG signals modulated with a high-pass (3 dB, 0.5 Hz) and a low-pass (50 Hz) analog filter and manually assessed. For both LD and DD conditions, continuous recordings were analyzed and time spent in each vigilance state was expressed as a percentage of the total recording time over various intervals (1–24 h). All DD recordings were started after at least 7 d of constant conditions. The mean duration of individual bouts of vigilance states was analyzed for the 12-h light/subjective day and 12-h dark/subjective night periods, and between ZT6 and ZT12 on baseline day and following 6 h of SD. The total amount of NREMS during SD was calculated as well as the latency to the first >25 epochs (100 s) of NREMS after 6 h of SD. Two mice from the SCN^Con^ group were excluded from the sleep analysis (*n* = 1 LD and DD; and *n* = 1 from DD only) because of the lack of sleep-wake data. Spectral analysis was computed for consecutive 4-s epochs by a fast Fourier transform (frequency range: 0.5−49.80 Hz; resolution 0.24 Hz; Hanning window function) on the EEG signal for wakefulness, NREMS and REMS. Genotypic differences were determined in DD over a complete circadian cycle, and expressed as either absolute EEG power or a percentage of total EEG power (i.e., relative EEG power) within all vigilance states for each mouse. Epochs containing EEG artefacts were discarded from the analyses. The time course of EEG δ activity (1–4 Hz) during NREMS was also computed in 2-h bins during LD and DD and after 6-h SD and presented as absolute EEG power and/or relative (i.e., as a percentage of the mean) to 24-h baseline for each mouse.

### Sleep deprivation and novel object recognition (NOR) test

Mice were recorded for a 24-h baseline day followed by 6-h SD and a further 18-h recovery sleep. SD (ZT0–ZT6) involved gentle procedures, i.e., introduction of novel objects such as nesting material, “fun tubes” and an initial cage change. NOR was tested in dim red light (<10 lux) between ZT20 and ZT22, in a red Perspex box measuring 50 × 50 × 50 cm with an overhead camera (Logitech Carl Zeiss Tessar HD 1080P) placed above the arena. The mice were habituated to the arena without objects for 10 min, followed by an initial familiarization session 24 h later where they were exposed to two identical objects for 10 min (plain or patterned Perspex objects, e.g., square, pyramid, oval, egg-cup all of similar sizes). After 24 h, the mice were re-tested with one of the objects being replaced by a novel object of similar size. Animals are assumed to have remembered the familiar object if they spend significantly more time investigating the novel object during the test phase. Investigation was considered when the mouse nose-pointed within 1 cm of the objects, but was not included if the mouse was climbing on the objects. The discrimination index (DI) was calculated as the difference between the time spent exploring the novel and familiar objects divided by the total time spent exploring the two objects [DI = (T_N_ – T_F_)/(T_N_ + T_F_)]. The time animals spent exploring each object in both the familiarization and test sessions were analyzed offline from the video recordings, using software designed by the laboratory of Prof W. Wisden, Imperial College, London, United Kingdom (Yu et al., 2014) with the experimenter blind to the genotype of the animal.

### Experimental design and statistical analysis

Analyses were conducted in Prism version 9.1.2 for macOS X (GraphPad software). One-way or two-way ANOVA, with repeated measures (RM) where relevant, and with *post hoc* Tukey's, Dunnett's, or Sidak's multiple comparisons tests were used to compare changes in sleep/wake parameters across genotypes. Where sphericity of the data was not assumed a Geisser–Greenhouse's ε correction was used (and so fractional degrees of freedom values are used to compute a *p* value), as recommended in Prism. Data from the running-wheels were analyzed using ClockLab (Actimetrics Inc.), running within MATLAB (MathWorks). Circadian period (χ^2^ periodogram analysis; unpaired Student's *t* test between WT and SCN^Cry1^ mice) and mean DD activity profiles were calculated for each animal, where activity was averaged over 8–10 d of activity and organized into 0.1-h bins. Comparisons of sleep-wake bouts, duration and frequency were made using ANOVA between genotypes and within genotype. In all cases, the experimental unit was an individual mouse. Male mice were used to avoid the confounding effect of oestrus cycles on circadian behavior patterns observed in female mice. Following attrition because of technical difficulties, the three treatment-group sizes were WT *n* = 5, SCN^Con^ *n* = 7, and SCN^Cry1^ *n* = 7. Given the variance of our measures, these sample sizes would yield a statistical power of 90–95% (G*Power 3.1, University of Dusseldorf, Dusseldorf, Germany).

## Results

### Local expression of Cry1 in the suprachiasmatic hypothalamus initiates circadian wheel-running behavior in clockless mice

**Figure 2.**
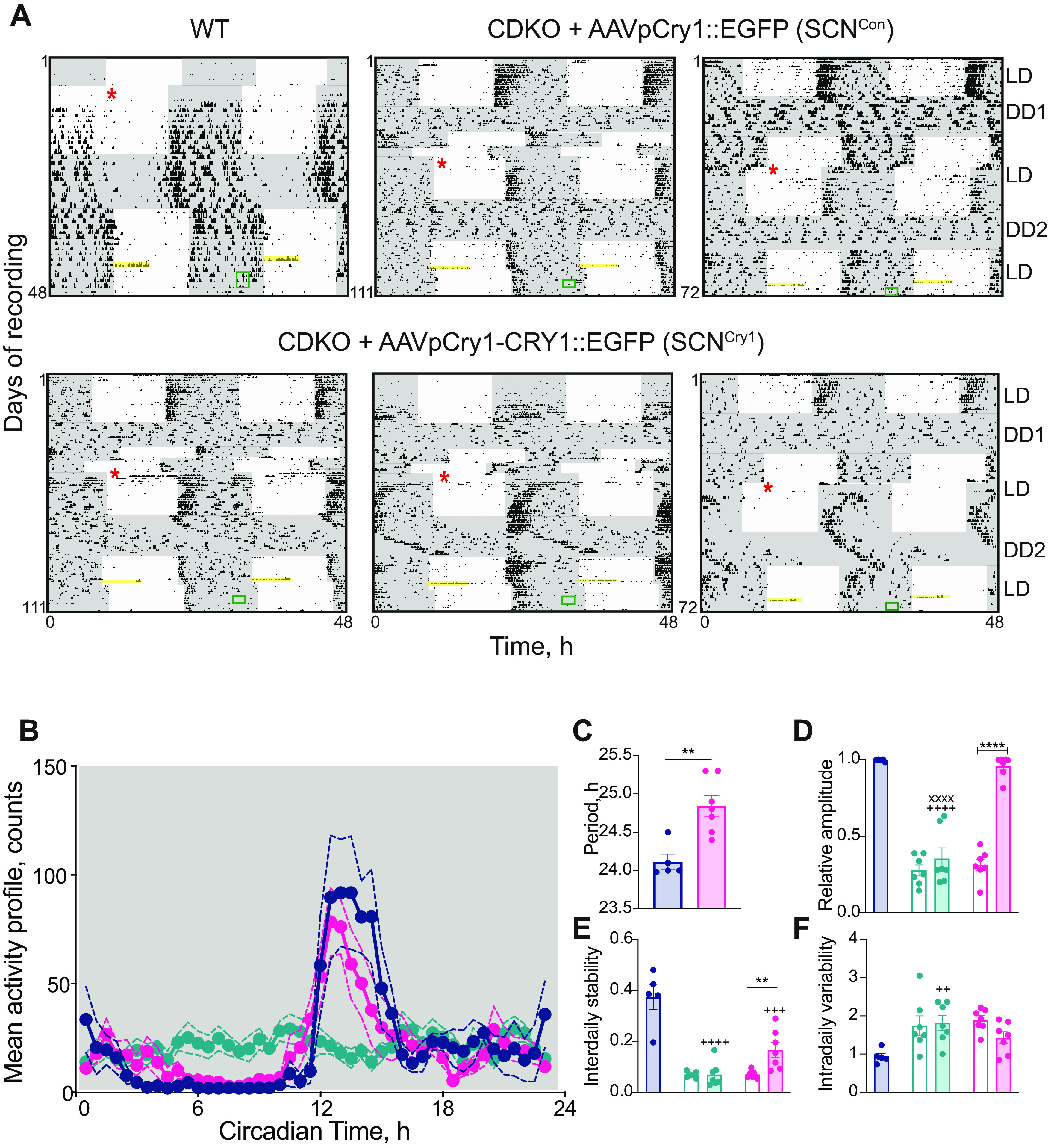
Local expression of Cry1 in the suprachiasmatic hypothalamus initiates circadian wheel-running behavior in clockless mice. ***A***, Double-plotted wheel-running traces from a WT (top left), two SCN^Con^ (middle and top right), and three representative traces from SCN^Cry1^ mice (bottom panel). Gray shaded areas represent darkness (LD; DD) before (DD1) and after (DD2) surgery (denoted by the red asterisk). The CDKO mice were arrhythmic presurgery, and the SCN^Con^ mice remained arrhythmic postsurgery, whereas SCN^Con^ mice showed significant circadian rhythmicity postsurgery. The yellow bar highlights the 6-h SD and the green box the timing of the NOR testing. ***B***, Mean activity (±SEM) counts over a circadian cycle in WT (*n* = 5; blue), SCN^Con^ (*n* = 7; green), and SCN^Cry1^ (*n* = 7; magenta); 2×RMANOVA: interaction: *F*_(90,630)_ = 4.0, *p* < 0.0001; genotype: *F*_(2,14)_ = 0.09, *p* = 0.9138; time: *F*_(45,630)_ = 10.77, *p* < 0.0001). ***C–F***, Mean (±SEM) and individual values of period (***C***), relative amplitude (***D***), interdaily stability (***E***), and intradaily variability (***F***) in WT (*n* = 5; blue), SCN^Con^ (*n* = 6; green), and SCN^Cry1^ (*n* = 7; magenta). Open bars are presurgery, shaded bars postsurgery (period: two-tailed unpaired Student's *t* test: *t*_(10)_ = 4.03; *p* < 0.005; 2×RMANOVA comparison of presurgery and postsurgery measures between SCN^Con^ and SCN^Cry1^ genotypes: relative amplitude: interaction: *F*_(1,12)_ = 29.6 *p* = 0.0002, genotype: *F*_(1,12)_ = 70.35 *p* < 0.0001; surgery: *F*_(1,12)_ = 56.17 *p* < 0.0001; interdaily stability: interaction: *F*_(1,12)_ = 7.0 *p* = 0.021, genotype: *F*_(1,12)_ = 6.4 *p* = 0.0265; surgery: *F*_(1,12)_ = 8.19 *p* = 0.0143; intradaily variability: interaction: *F*_(1,12)_ = 2.15 *p* = 0.17, genotype: *F*_(1,12)_ = 0.036 *p* = 0.852; surgery: *F*_(1,12)_ = 0.36 *p* = 0.5586; 1×ANOVA comparing WT vs postsurgery of SCN^Con^ and SCN^Cry1^ genotype: relative amplitude: *F*_(2,16)_ = 59.9 *p* < 0.0001, *post hoc* Tukey's multiple comparisons tests WT vs SCN^Con^ *p* < 0.0001, WT vs SCN^Cry1^ *p* = 0.8419, SCN^Cry1^ vs SCN^Con^ *p* < 0.0001; interdaily stability: *F*_(2,16)_ = 23.15 *p* < 0.0001, *post hoc* Tukey's multiple comparisons tests WT vs SCN^Con^ *p* < 0.0001, WT vs SCN^Cry1^ *p* = 0.0006, SCN^Cry1^ vs SCN^Con^ *p* = 0.11; intradaily variability: *F*_(2,16)_ = 6.3 *p* = 0.0098, *post hoc* Tukey's multiple comparisons tests WT vs SCN^Con^ *p* = 0.0072, WT vs SCN^Cry1^ *p* = 0.1247, SCN^Cry1^ vs SCN^Con^ *p* = 0.2765; **p < 0.01, ***p < 0.0001; ++*p* < 0.0001, +++*p* < 0.001, ++++*p* < 0.01 vs WT; xxxx*p* < 0.0001 vs SCN^Cry1^).

Local, bilateral AAV-mediated nuclear expression of Cry1::EGFP in the suprachiasmatic hypothalamus of *Cry1/Cry2*-null mice was evident from the EGFP tag (Fig. 1*B–D*). It was limited to the SCN and the immediately surrounding hypothalamus, in some cases extending into the anterior hypothalamic area and paraventricular nucleus (PVN) and/or posteriorly to the retrochiasmatic area and/or anteriorly to the medial preoptic area (Fig. 1*E*). Cry1 did not extend into the dorsal or lateral hypothalamus, and there was no consistent pattern of extra-SCN expression between animals: the SCN was the only target common to all mice (see overlay in Fig. 1*E*). The total proportion of transfected cells within the paired SCN, together, was 46.5 ± 6.5% (*n* = 7; range 24–74%), as assessed by the ratio of EGFP-positive cells to DAPI-positive cells using ImageJ, which is in agreement with our previous studies (Maywood et al., 2018; Brancaccio et al., 2019). WT mice exhibited robust circadian cycles of wheel-running behavior, whereas *Cry1,2*-null mice targeted with EGFP (SCN^Con^) were arrhythmic in DD before and after surgery (Fig. 2*A*). In contrast, previously arrhythmic SCN^Cry1^ mice exhibited robust circadian wheel-running behavior following expression of Cry1::EGFP, as demonstrated previously (Maywood et al., 2018). The mean postsurgery activity profile between groups shows the significant circadian rhythmicity in both the WT and SCN^Cry1^ mice, whereas activity in SCN^Con^ mice was distributed evenly across the circadian cycle, phase-referenced to the prior LD cycle (Fig. 2*B*). Furthermore, the period of 24.8 ± 0.2 h (*n* = 7; Fig. 2*C*) in SCN^Cry1^ mice was significantly longer than in WT (*n* = 5; 24.1 ± 0.1 h) and diagnostic of a Cry1-driven TTFL (van der Horst et al., 1999; Maywood et al., 2018). Finally, non-parametric analysis of locomotor activity confirmed the excellent circadian organization of WT mice, and its disorganization in SCN^Con^ mice, which had low relative amplitude and stability and high variability (Fig. 2*D–F*). In contrast, previously arrhythmic SCN^Cry1^ mice showed robust circadian behavior after surgery, with significantly improved organization comparable to WT mice, and a non-significant trend for lower intradaily variability (see legend to Fig. 2 for statistical analyses).

### Rescue of circadian sleep/wake patterning in SCN^Cry1^ mice

**Figure 3.**
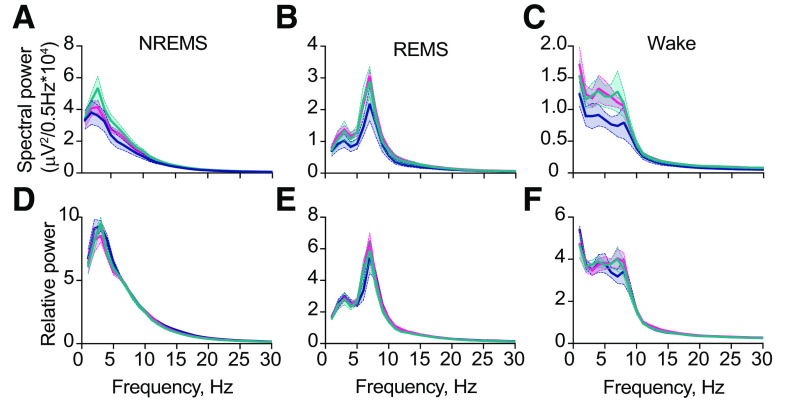
Local expression of Cry1 in the suprachiasmatic hypothalamus organizes circadian sleep/wake patterning. ***A–C***, Mean baseline raw EEG spectral power in NREMS (***A***), REMS (***B***), and wake (***C***) over a circadian cycle (DD; 2×RMANOVA: NREMS: interaction: *F*_(120,900)_ = 1.0, *p* = 0.43, genotype: *F*_(2,15)_ = 1.12, *p* = 0.3555, frequency: *F*_(1.6,23.4)_ = 143.2, *p* < 0.0001; REMS: interaction: *F*_(120,900)_ = 1.02, genotype: *F*_(2,15)_ = 1.25, *p* = 0.3151, frequency: *F*_(1.9,25.9)_ = 98.69, *p* < 0.0001 *p* = 0.44; wake: interaction: *F*_(120,900)_ = 0.68, *p* > 0.99, genotype: *F*_(2,15)_ = 1.52, *p* = 0.2506, frequency: *F*_(1.6,23.4)_ = 55.74, *p* < 0.0001). ***D–F***, Mean baseline relative EEG spectral power (relative to total power) in NREMS (***D***), REMS (***E***), and wake (***F***) over a circadian cycle (DD; 2×RMANOVA: NREMS: interaction: *F*_(120,900)_ = 0.52, *p* > 0.99, genotype: *F*_(2,15)_ = 1.28, *p* = 0.3064, frequency: *F*_(1.6,26.1)_ = 368.1, *p* < 0.0001; REMS: interaction: *F*_(120,900)_ = 0.91, *p* = 0.7926, genotype: *F*_(2,15)_ = 0.4, *p* = 0.6768, frequency: *F*_(2.3,31.9)_ = 214.0, *p* < 0.0001; wake: interaction: *F*_(120,900)_ = 0.32, *p* > 0.99, genotype: *F*_(2,15)_ = 0.60, *p* = 0.5615, frequency: *F*_(1.6,24.7)_ = 160.5, *p* < 0.0001). WT (*n* = 5), blue; SCN^Con^ (*n* = 6), green; SCN^Cry1^ (*n* = 7), magenta.

Initiation of circadian control of wheel-running behavior by AAV-mediated genetic complementation made it possible to explore the degree of control to sleep mediated by molecular competence in the suprachiasmatic hypothalamus. EEG spectral analysis showed that under DD, in the absence of any masking or other effects of light, the different vigilance states exhibited their characteristic neurophysiological features (Fig. 3). Moreover, the genotype had no significant effect on these parameters, confirming that the absence of Cry proteins does not affect the core molecular and neural machinery that generates the states of wakefulness, REMS, and NREMS

**Figure 4.**
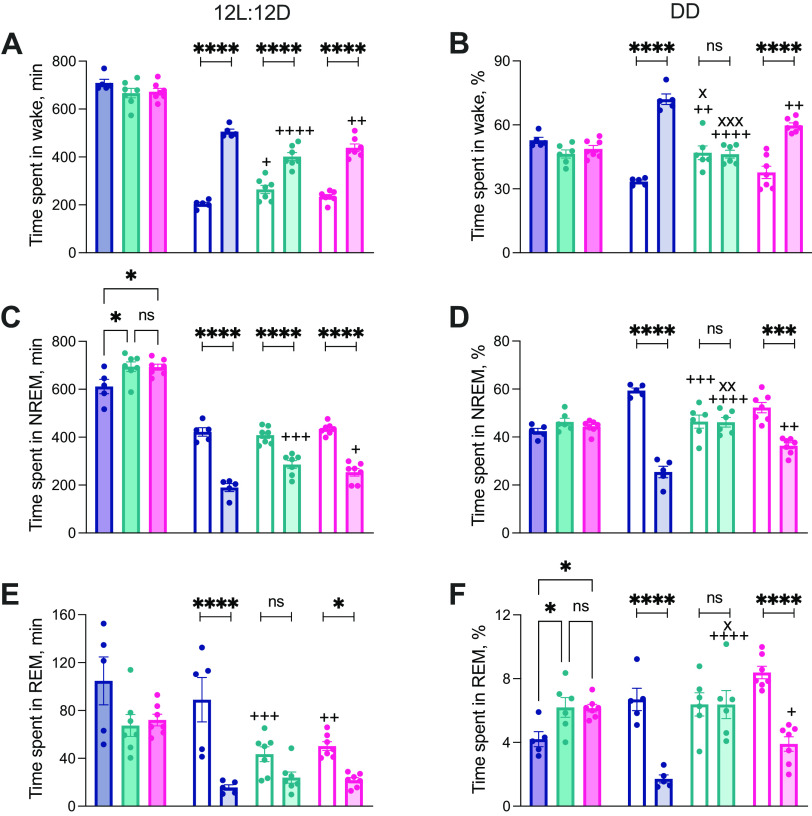
Local expression of Cry1 in the suprachiasmatic hypothalamus organizes circadian sleep/wake patterning. ***A***, ***C***, ***E***, Time spent in wakefulness (***A***), NREMS (***C***), and REMS (***E***) in 12hL:12D (minutes; mean ± SEM and individual points). Dark shaded bars on the left-hand side (LHS) show time over 24 h (see Results; 1×ANOVA: NREMS LD 24 h, *post hoc* Tukey's multiple comparisons test +*p* < 0.05 vs WT), with the 12-h light (clear) and 12-h dark (lightly shaded) bars on the right-hand side (RHS). There is clear day (rest)/night (active) organization in all vigilance states in both WT and SCN^Cry1^ mice but only in wake and NREMS for the SCN^Con^ mice (2×RMANOVA: wake: interaction: *F*_(2,16)_ = 11.94, *p* = 0.0007, genotype: *F*_(2,16)_ = 1.615, *p* = 0.2297; time: *F*_(1,16)_ = 256.4, *p* = 0.0007, *post hoc* Sidak's multiple comparisons test day-night: WT, SCN^Con^, and SCN^Cry1^ all *p* < 0.0001, *post hoc* Tukey's multiple comparisons test, day: WT vs SCN^Con^ *p* = 0.0174, WT vs SCN^Cry1^ *p* = 0.3224, SCN^Con^ vs SCN^Cry1^ *p* = 0.2648, night: WT vs SCN^Con^ *p* < 0.0001, WT vs SCN^Cry1^ *p* = 0.0085, SCN^Con^ vs SCN^Cry1^ *p* = 0.1521; NREMS: interaction: *F*_(2,16)_ = 6.5, *p* = 0.0085, genotype: *F*_(2,16)_ = 4.144, *p* = 0.0355; time: *F*_(1,16)_ = 214.1, *p* < 0.000, *post hoc* Sidak's multiple comparisons test day-night: WT, SCN^Con^, and SCN^Cry1^ all *p* < 0.0001, *post hoc* Tukey's multiple comparisons test, day: WT vs SCN^Con^ *p* = 0.8075, WT vs SCN^Cry1^ *p* = 0.8383, SCN^Con^ vs SCN^Cry1^ *p* = 0.4021, night: WT vs SCN^Con^ *p* = 0.0003, WT vs SCN^Cry1^ *p* = 0.017, SCN^Con^ vs SCN^Cry1^ *p* = 0.2505; REMS: interaction: *F*_(2,16)_ = 9.1, *p* = 0.0023, genotype: *F*_(2,16)_ = 0.0821, *p* = 0.0821; time: *F*_(1,16)_ = 59.79, *p* < 0.0001, *post hoc* Sidak's multiple comparisons test day-night: WT and SCN^Cry1^ *p* < 0.0001, SCN^Con^ *p* = 0.1026, *post hoc* Tukey's multiple comparisons test, day: WT vs SCN^Con^ *p* = 0.0174, WT vs SCN^Cry1^ *p* = 0.3224, SCN^Con^ vs SCN^Cry1^ *p* = 0.2648, night: WT vs SCN^Con^ *p* < 0.0001, WT vs SCN^Cry1^ *p* = 0.0085, SCN^Con^ vs SCN^Cry1^ *p* = 0.1521). ***B***, ***D***, ***F***, Time spent in wakefulness (***B***), NREMS (***D***), and REMS (***E***) in DD (% time; mean ± SEM and individual points). Dark shaded bars on LHS show time over a circadian cycle (1×ANOVA: REMS DD, *post hoc* Tukey's multiple comparisons test +*p* < 0.05 vs WT), with the circadian day/rest phase (clear) and circadian night/active phase (lightly shaded) bars on the RHS. There is clear circadian day (rest)/night (active) organization in all vigilance states in both WT and SCN^Cry1^ mice but only in the SCN^Con^ mice (2×RMANOVA: wake: interaction: *F*_(2,15)_ = 35.74, *p* < 0.0001, genotype: *F*_(2,15)_ = 3.106, *p* = 0.0744; time: *F*_(1,15)_ = 117.2, *p* < 0.0001, *post hoc* Sidak's multiple comparisons test day-night: WT and SCN^Cry1^ *p* < 0.0001, SCN^Con^ *p* = 0.9957, *post hoc* Tukey's multiple comparisons test, day: WT vs SCN^Con^ *p* = 0.0013, WT vs SCN^Cry1^ *p* = 0.4087, SCN^Con^ vs SCN^Cry1^ *p* = 0.0175, night: WT vs SCN^Con^ *p* < 0.0001, WT vs SCN^Cry1^ *p* = 0.0023, SCN^Con^ vs SCN^Cry1^ *p* = 0.0005; NREMS: interaction: *F*_(2,15)_ = 25.3, *p* < 0.0001, genotype: *F*_(2,15)_ = 2.196, *p* = 0.1457; time: *F*_(1,15)_ = 80.98, *p* < 0.0001, *post hoc* Sidak's multiple comparisons test day-night: WT *p* < 0.0001, SCN^Cry1^ *p* = 0.0002, SCN^Con^ *p* = 0.9996, *post hoc* Tukey's multiple comparisons test, day: WT vs SCN^Con^ *p* = 0.0005, WT vs SCN^Cry1^ *p* = 0.0522, SCN^Con^ vs SCN^Cry1^ *p* = 0.1075, night: WT vs SCN^Con^ *p* < 0.0001, WT vs SCN^Cry1^ *p* = 0.0021, SCN^Con^ vs SCN^Cry1^ *p* = 0.0036; REMS: interaction: *F*_(2,15)_ = 19.67, *p* < 0.0001, genotype: *F*_(2,15)_ = 4.735, *p* = 0.0255; time: *F*_(1,15)_ = 78.41, *p* < 0.0001, *post hoc* Sidak's multiple comparisons test day-night: WT and SCN^Cry1^ *p* < 0.0001, SCN^Con^ *p* = 0.9999, *post hoc* Tukey's multiple comparisons test, day: WT vs SCN^Con^ *p* = 0.9361, WT vs SCN^Cry1^ *p* = 0.1428 SCN^Con^ vs SCN^Cry1^ *p* = 0.0542, night: WT vs SCN^Con^ *p* < 0.0001, WT vs SCN^Cry1^ *p* = 0.0432, SCN^Con^ vs SCN^Cry1^ *p* = 0.014); *p < 0.05, ***p < 0.001, ****p < 0.0001 within genotype; +*p* < 0.05, ++*p* < 0.01, +++*p* < 0.001, ++++*p* < 0.0001 versus WT; x*p* < 0.05, xx*p* < 0.01, xxx*p* < 0.001 versus SCN^Cry1^. WT (*n* = 5), blue; SCN^Con^ (*n* = 6), green; SCN^Cry1^ (*n* = 7), magenta.

The total amount of wake did not vary between groups under LD (24 h) or DD (circadian cycle; Fig. 4*A*,*B*; wake LD: 1×ANOVA *F*_(2,16)_ = 1.615, *p* = 0.2298; wake DD: 1×ANOVA *F*_(2,15)_ = 3.26, *p* = 0.0669), but compared with WT controls, both SCN^Con^ and SCN^Cry1^ mice exhibited more NREMS under LD, as reported (Wisor et al., 2002; NREMS LD: 1×ANOVA *F*_(2,16)_ = 4.7, *p* = 0.024; Tukey's multiple comparisons test: WT vs SCN^Con^ *p* = 0.0347, WT vs SCN^Cry1^ *p* = 0.0397, SCN^Con^ vs SCN^Cry1^ *p* = 0.9969; NREMS DD: 1×ANOVA *F*_(2,15)_ = 2.2, *p* = 0.15). In DD, but not LD, both SCN^Con^ and SCN^Cry1^ mice showed a small elevation in REMS (REMS LD: 1×ANOVA *F*_(2,16)_ = 2.9, *p* = 0.08; REMS DD: 1×ANOVA *F*_(2,15)_ = 5.7, *p* = 0.015; Tukey's multiple comparisons test: WT vs SCN^Con^ *p* = 0.0245, WT vs SCN^Cry1^ *p* = 0.0232, SCN^Con^ vs SCN^Cry1^ *p* = 0.9967; Fig. 4*C–F*). These likely represent Cry-dependent traits independent of the SCN.

We then examined the temporal distribution of sleep/wake. Under LD, WT mice showed appropriate nocturnal wakefulness and more NREMS and REMS in daytime (Fig. 4*A*,*C*,*E*). Equally, both *Cry1, Cry2*-null groups, SCN^Con^ and SCN^Cry1^, had clearly defined light/dark differences in wake and NREMS (Fig. 4*A*,*C*), whereas SCN^Con^ mice did not show significant light/dark differences in REMS in LD (Fig. 4*E*). Despite the light/dark organization of the sleep/wake patterns, both the SCN^Con^ and SCN^Cry1^ mice spent significantly less time in wake in the dark phase compared with WT (Fig. 4*A*,*C*,*E*; as assessed by *post hoc* Sidak's and Tukey's multiple comparisons tests within genotype and between genotypes, respectively, where there was a significant interaction and/or genotype effect in a 2×RMANOVA; see legend to Fig. 4 for details).

Under DD, WT mice again showed clear differences between subjective day (rest phase; higher levels of NREMS and REMS) and subjective night (active phase; more wake; Fig. 4*B*,*D*,*F*). In contrast, under DD, the SCN^Con^ mice showed no significant circadian patterning to vigilance states (Fig. 4*B*,*D*,*F*; no significant rest/active differences within genotype assessed using 2×RMANOVA with *post hoc* Sidak's multiple comparisons tests; see legend to Fig. 4 for statistical analyses). Unlike in LD, where the light imposed a level of organization on the sleep/wake profiles, under DD the SCN^Con^ mice spent significantly more time in wake (Fig. 4*B*) and less time in NREMS in the circadian day (Fig. 4*D*), and significantly less time in wake and more NREMS and REMS in circadian night, compared with WT (Fig. 4*B*,*D*,*F*; as assessed by *post hoc* Tukey's multiple comparisons between groups where there was a significant interaction and/or genotype effect in a 2×RMANOVA; see legend to Fig. 4 for details). In contrast, SCN^Cry1^ mice showed robust circadian organization to the sleep-wake cycle, with clear subjective day and night differences comparable to those of WT controls across all vigilance states. Nevertheless, these mice did spend significantly less time in wake and more time in NREMS and REMS during the circadian night compared with WT (Fig. 4*B*,*D*,*F*; as assessed by *post hoc* Tukey's multiple comparisons between genotypes where there was a significant interaction and/or genotype effect in a 2×RMANOVA; see legend to Fig. 4 for details). The loss of circadian organization to sleep in SCN^Con^ mice was therefore reversed in SCN^Cry1^ mice, enabling the *de novo* establishment of a more WT-like organization to the diurnal/circadian patterning to sleep/wake.

**Figure 5.**
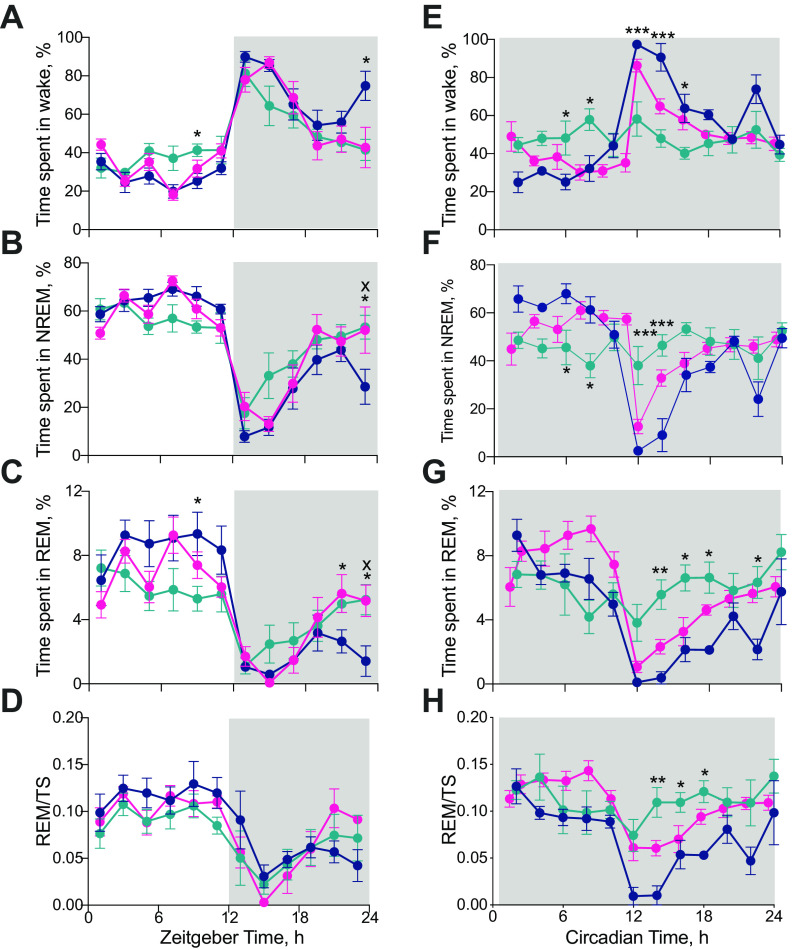
Local expression of Cry1 in the suprachiasmatic hypothalamus organizes circadian sleep/wake patterning. ***A–D***, 2-h profiles (mean ± SEM) of wakefulness (***A***), NREMS (***B***), REMS (***C***), and REMS/TS (***D***) in WT (*n* = 5; blue), SCN^Con^ (*n* = 7; green), and SCN^Cry1^ (*n* = 7; magenta) under a 12/12 h light/dark photoschedule (2×RMANOVA wake: interaction: *F*_(22,176)_ = 2.23, *p* = 0.0022, genotype: *F*_(2,16)_ = 0.9038, *p* = 0.4247; time: *F*_(11,176)_ = 30.82, *p* < 0.0001; NREMS: interaction: *F*_(22,176)_ = 2.0, *p* = 0.0072, genotype: *F*_(2,16)_ = 1.99, *p* = 0.1673; time: *F*_(11,176)_ = 31.17, *p* < 0.0001; REMS: interaction: *F*_(22,176)_ = 2.85, *p* < 0.0001, genotype: *F*_(2,16)_ = 0.17, *p* = 0.8497; time: *F*_(11,176)_ = 26.4, *p* < 0.0001; REM/TS: interaction: *F*_(22,176)_ = 0.93, *p* = 0.55; genotype: *F*_(2,16)_ = 0.65, *p* = 0.5377; time: *F*_(5,75)_ = 12.51, *p* < 0.0001; *post hoc* Tukey's multiple comparison tests *p < 0.05, **p < 0.01, ***p < 0.001 WT vs SCN^Con^; x*p* < 0.05, xx*p* < 0.01 WT vs SCN^Cry1^; +*p* < 0.05 SCN^Con^ vs SCN^Cry1^). ***E–H***, Two circadian hour profiles (mean ± SEM) of wakefulness (***E***), NREMS (***F***), REMS (***G***), and REMS/TS (***H***) in WT (*n* = 5; blue), SCN^Con^ mice (*n* = 6; green), and SCN^Cry1^ (*n* = 7; magenta) under free-running constant conditions [note, not possible to do statistics including SCN^Cry1^ as these mice have a different endogenous free-running period and so a different timescale] (2×RMANOVA wake: interaction: *F*_(11,99)_ = 7.6, *p* < 0.0001, genotype: *F*_(1,9)_ = 5.073, *p* = 0.0508; time: *F*_(11,99)_ = 9.75, *p* < 0.0001; NREMS: interaction: *F*_(11,99)_ = 7.5, *p* < 0.0001, genotype: *F*_(1,9)_ = 2.5, *p* = 0.1481; time: *F*_(11,99)_ = 9.3, *p* < 0.0001; REMS: interaction: *F*_(11,99)_ = 4.6, *p* < 0.0001, genotype: *F*_(1,9)_ = 5.26, *p* = 0.0474; time: *F*_(11,99)_ = 7.5, *p* < 0.0001; REM/TS: interaction: *F*_(11,99)_ = 2.8, *p* = 0.0033, genotype: *F*_(1,9)_ = 5.84, *p* = 0.0388; time: *F*_(11,99)_ = 6.68, *p* < 0.0001; *post hoc* Tukey's multiple comparison test *, **, *** *p* < 0.05, 0.01, 0.001 WT vs SCN^Con^). Gray shaded area represents darkness.

A finer-grained, 2-h resolution, analysis of the 24-h distribution of sleep/wake emphasized further the effects on sleep-wake patterning of global Cry1,2 deficiency and local restoration of clock function in the SCN region (Fig. 5; as assessed by *post hoc* Tukey's multiple comparisons tests between genotypes where there was either a significant interaction and/or genotype effect in a 2×RMANOVA; see legend to Fig. 5 for details). Under LD, all groups exhibited a daily pattern of vigilance states, but with a reduced amplitude in the SCN^Con^ mice, likely reflecting their poor behavioral entrainment to, and/or masking by, the photoschedule (Fig. 5*A–D*). Furthermore, in the second half of the light phase (ZT6–ZT12), SCN^Con^ mice had significantly more wakefulness (+50 min) and less NREMS (−40 min) compared with WT mice. Expression of Cry1 in the SCN region reversed these deficits (1×ANOVA ZT6–ZT12: wake amount: *F*_(2,16)_ = 8.3, *p* < 0.005; *post hoc* Tukey's multiple comparison test: WT vs SCN^Con^ *p* = 0.003; WT vs SCN^Cry1^ *p* = 0.36; SCN^Cry1^ vs SCN^Con^ *p* = 0.035; NREMS amount: *F*_(2,16)_ = 7.2, *p* = 0.0059; *post hoc* Tukey's multiple comparison test: WT vs SCN^Con^ *p* = 0.007; WT vs SCN^Cry1^ *p* = 0.55; SCN^Cry1^ vs SCN^Con^ *p* = 0.036). Conversely, at the end of the dark phase, WT mice showed more wake and less NREMS and REMS than both CDKO groups (Fig. 5*A–C*). Finally, the amount of REM as a proportion of total sleep (TS = NREMS + REMS) was high in day and low at night in WT mice and this pattern was replicated by SCN^Con^ and SCN^Cry1^ (Fig. 5*D*). Under LD, therefore, loss of Cry proteins altered the temporal distribution of vigilance states across the 24 h, and this was partially restored by Cry1 expression focused on the SCN.

The group differences between WT and SCN^Con^ mice were amplified in DD (Fig. 5*E–H*; see legend to Fig. 5 for 2×RMANOVA analyses between genotypes, although not possible to do statistics including the group SCN^Cry1^ as these mice have a different endogenous free-running period and so a different timescale). Whereas WT exhibited robust circadian patterning, SCN^Con^ mice failed to show any significant organization of wake, NREMS or REMS across the circadian cycle (Fig. 5*E–G*). Similarly, in WT mice the amount of REMS as a proportion of TS, which under DD is a measure of circadian control independent of changes in the absolute amount of wakefulness, was highly circadian, whereas it was not in SCN^Con^ mice (REM/TS: WT: 1×ANOVA *F*_(11,48)_ = 5.8, *p* < 0.0001; SCN^Con^: *F*_(11,60)_ = 0.9; Fig. 5*H*). Expression of Cry1 had a marked restorative effect on sleep/wake patterns in DD. The SCN^Cry1^ mice showed a more WT-like organization of the sleep/wake cycle across circadian day and night (Fig. 5*E–G*), although this rescue was not complete as they did have slightly less wakefulness (∼12%) and more NREMS (∼10%) and REMS (∼2%) in the circadian night (CT12–CT24) compared with WT. Levels of REMS/TS were higher overall in SCN^Cry1^ mice, but they nevertheless showed a significant circadian rhythm in the distribution of REMS/TS over the circadian cycle (REMS/TS: SCN^Cry1^: 1×ANOVA *F*_(12,78)_ = 7.72, *p* < 0.0001; Fig. 5*H*) and, as with the WT mice but not the SCN^Con^ mice, had significantly lower overall levels of REMS/TS in circadian night (total CT12–CT24) than in circadian day (total CT0–CT12; total REMS/TS in circadian day/circadian night: 2×RMANOVA: interaction: *F*_(2,15)_ = 21.12, *p* < 0.0001, genotype: *F*_(2,15)_ = 5.18, *p* = 0.0195; time: *F*_(1,15)_ = 31.94, *p* < 0.0001; *post hoc* Sidak's multiple comparison test: total in circadian day vs total in circadian night: WT *p* = 0.0056; SCN^Con^ *p* = 0.38; SCN^Cry1^ *p* < 0.0001). Together, the LD and DD analyses confirm the interaction between light and the circadian system in organizing sleep/wake cycles (Tsai et al., 2009), and suggest that the suprachiasmatic clockwork has a sleep-promoting/wake-suppressing effect in the second half of the light/rest phase of the LD and DD cycles (ZT/CT6–CT12). Nevertheless, in the absence of a lighting cycle, a molecularly competent SCN is able to impose a circadian distribution of sleep-wake patterns in an otherwise clockless mouse.

### Consolidation of disrupted sleep/wake architecture in SCN^Cry1^ mice

**Figure 6.**
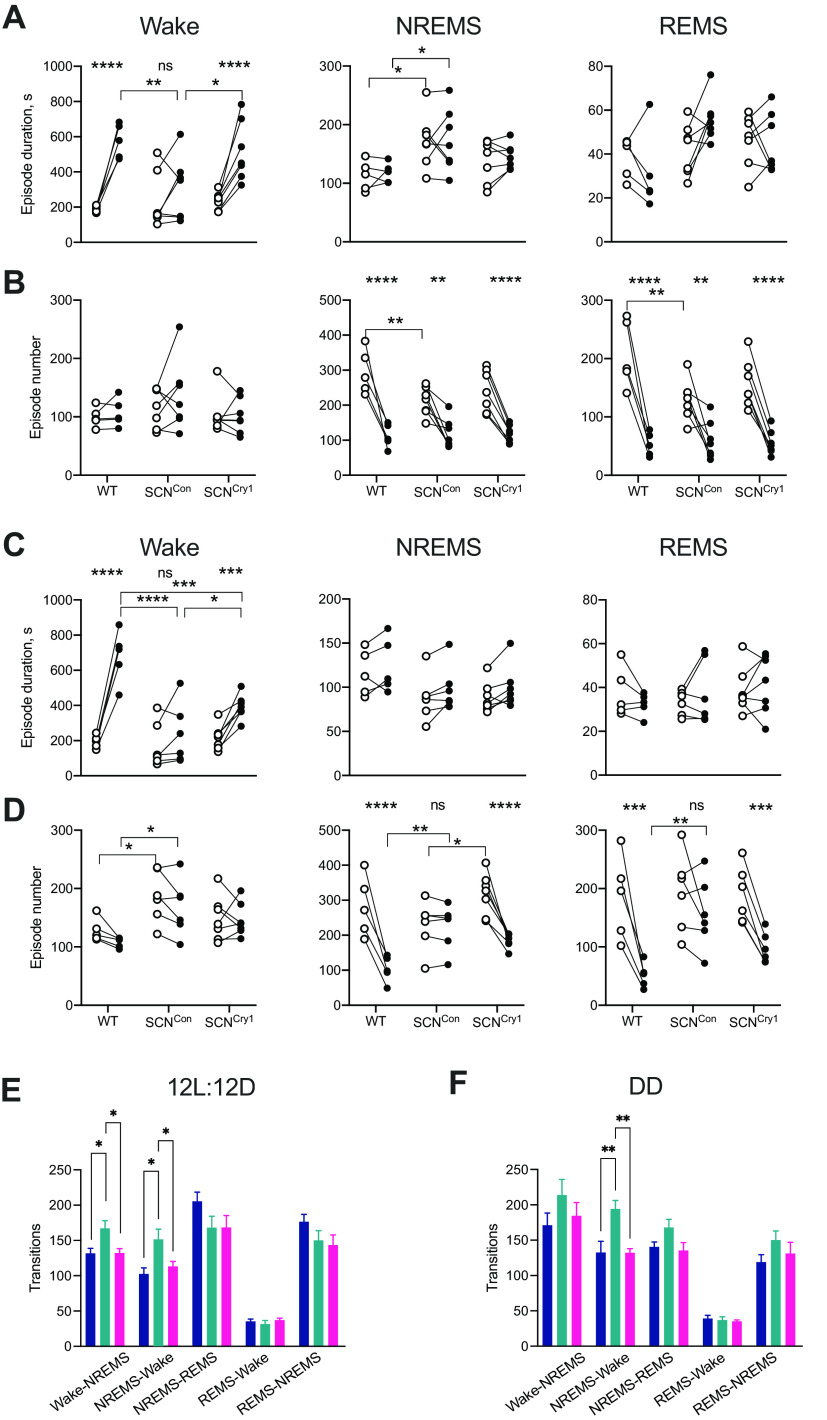
Local expression of Cry1 in the suprachiasmatic hypothalamus consolidates sleep/wake architecture. ***A***, Duration (seconds) of bouts in wakefulness, NREM, and REM in 12 h light (open circles) and 12 h dark (closed circles) in WT (*n* = 5, blue), SCN^Con^ (*n* = 7, green), and SCN^Cry1^ (*n* = 7, magenta) mice in entrained (LD) conditions. Whereas the WT and SCN^Cry1^ mice have a significant increase in wake duration during the dark/active compared with the light/inactive phase, the SCN^Con^ animals have significantly fewer nocturnal bouts and so do not have a significant LD organization [episode duration: 2×RMANOVA wake: interaction: *F*_(2,16)_ = 8.5, *p* = 0.003; genotype: *F*_(2,16)_ = 1.97, *p* = 0.1715; time: *F*_(1,16)_ = 61.38, *p* < 0.0001; *post hoc* multiple comparisons tests: Sidak's (LD): WT *p* < 0.0001; SCN^Con^ *p* = 0.4397; SCN^Cry1^ *p* < 0.0001; and Tukey's (genotype): light: WT vs SCN^Con^ *p* = 0.8393; WT vs SCN^Cry1^ *p* = 0.8368; SCN^Con^ vs SCN^Cry1^ *p* > 0.9999; dark: WT vs SCN^Con^ *p* = 0.0044; WT vs SCN^Cry1^ *p* = 0.7266; SCN^Con^ vs SCN^Cry1^ *p* = 0.0161; NREMS: interaction: *F*_(2,16)_ = 0.0665, *p* = 0.9359; genotype: *F*_(2,16)_ = 4.393, *p* = 0.0302; time: *F*_(1,16)_ = 0.4075, *p* = 0.5323; *post hoc* multiple comparisons tests: Sidak's (LD): WT *p* = 0.9798; SCN^Con^ *p* = 0.9991; SCN^Cry1^ *p* = 0.8986; and Tukey's (genotype): light: WT vs SCN^Con^ *p* = 0.026; WT vs SCN^Cry1^ *p* = 0.4817; SCN^Con^ vs SCN^Cry1^ *p* = 0.2106; dark: WT vs SCN^Con^ *p* = 0.037; WT vs SCN^Cry1^ *p* = 0.415; SCN^Con^ vs SCN^Cry1^ *p* = 0.3408; REMS: interaction: *F*_(2,16)_ = 3.185, *p* = 0.0685; genotype: *F*_(2,16)_ = 3.041, *p* = 0.076; time: *F*_(1,16)_ = 0.237, *p* = 0.633]. ***B***, Number of bouts of wakefulness, NREMS and REMS in 12 h light (open circles) and 12 h dark (closed circles) in WT (*n* = 5, blue), SCN^Con^ (*n* = 7, green), and SCN^Cry1^ (*n* = 7, magenta) mice in LD. All three genotypes have a significant LD organization of NREMS and REMS episodes, although the SCN^Con^ mice have fewer bouts of NREMS and REMS in the light period, i.e., the rest period [episode number: 2×RMANOVA wake: interaction: *F*_(2,16)_ = 0.635, *p* = 0.5427; genotype: *F*_(2,16)_ = 1.17, *p* = 0.335; time: *F*_(1,16)_ = 0.93, *p* = 0.341; NREMS: interaction: *F*_(2,16)_ = 3.688, *p* = 0.0482; genotype: *F*_(2,16)_ = 1.779, *p* = 0.2006; time: *F*_(1,16)_ = 99.36, *p* < 0.0001; *post hoc* multiple comparisons tests: Sidak's (LD): WT *p* < 0.0001; SCN^Con^ *p* = 0.0019; SCN^Cry1^ *p* < 0.0001; and Tukey's (genotype): light: WT vs SCN^Con^ *p* = 0.0091; WT vs SCN^Cry1^ *p* = 0.1234; SCN^Con^ vs SCN^Cry1^ *p* = 0.4282; dark: WT vs SCN^Con^ *p* = 0.9661; WT vs SCN^Cry1^ *p* = 0.9778; SCN^Con^ vs SCN^Cry1^ *p* = 0.9985; REMS: interaction: *F*_(2,16)_ = 5.573, *p* = 0.0146; genotype: *F*_(2,16)_ = 2.259, *p* = 0.1367; time: *F*_(1,16)_ = 115.3, *p* < 0.0001; *post hoc* multiple comparisons tests: Sidak's (LD): WT *p* < 0.0001; SCN^Con^ *p* = 0.0019; SCN^Cry1^ *p* < 0.0001; and Tukey's (genotype): light: WT vs SCN^Con^ *p* = 0.0023; WT vs SCN^Cry1^ *p* = 0.0543; SCN^Con^ vs SCN^Cry1^ *p* = 0.3507; dark: WT vs SCN^Con^ *p* = 0.9347; WT vs SCN^Cry1^ *p* = 0.9904; SCN^Con^ vs SCN^Cry1^ *p* = 0.969]. ***C***, Duration (seconds) of bouts in wakefulness, NREM, and REM in subjective day (open circles) and subjective night (closed circles) in WT (*n* = 5, blue), SCN^Con^ (*n* = 6, green), and SCN^Cry1^ (*n* = 7, magenta) mice in free-running conditions (DD). Both the WT and SCN^Cry1^ mice have a significant circadian organization to wake duration but the SCN^Con^ animals do not, because of significantly fewer wake episodes during the circadian night/active phase when compared with the WT and SCN^Cry1^ mice [episode duration: 2×RMANOVA wake: interaction: *F*_(2,16)_ = 26.33, *p* < 0.0001; genotype: *F*_(2,16)_ = 7.14, *p* = 0.0066; time: *F*_(1,16)_ = 104.4, *p* < 0.0001; *post hoc* multiple comparisons tests: Sidak's (LD): WT *p* < 0.0001; SCN^Con^ *p* = 0.3901; SCN^Cry1^ *p* = 0.0008; and Tukey's (genotype): light: WT vs SCN^Con^ *p* = 0.9564; WT vs SCN^Cry1^ *p* = 0.9421; SCN^Con^ vs SCN^Cry1^ *p* = 7902; dark: WT vs SCN^Con^ *p* < 0.0001; WT vs SCN^Cry1^ *p* = 0.0004; SCN^Con^ vs SCN^Cry1^ *p* = 0.0472; NREMS: interaction: *F*_(2,16)_ = 0.072, *p* = 0.9306; genotype: *F*_(2,16)_ = 2.17, *p* = 0.1485; time: *F*_(1,16)_ = 11.19, *p* = 0.044; REMS: interaction: *F*_(2,16)_ = 1.543, *p* = 0.2458; genotype: *F*_(2,16)_ = 0.5545, *p* = 0.5857; time: *F*_(1,16)_ = 0.089, *p* = 0.792]. ***D***, Number of bouts of wakefulness, NREMS and REMS in subjective day (open circles) and subjective night (closed circles) in WT (*n* = 5, blue), SCN^Con^ (*n* = 6, green), and SCN^Cry1^ (*n* = 7, magenta) mice in DD. In the absence of light, the SCN^Con^ mice no longer show a circadian organization in the number of NREMS and REMS episodes, with significantly more episodes in the dark/active period compared with the WT mice [episode number: 2×RMANOVA wake: interaction: *F*_(2,16)_ = 0.8968, *p* = 0.4266; genotype: *F*_(2,16)_ = 4.52, *p* = 0.0291; time: *F*_(1,16)_ = 4.714, *p* = 0.0464; *post hoc* multiple comparisons tests: Sidak's (LD): WT *p* = 0.29 569; SCN^Con^ *p* = 0.2809; SCN^Cry1^ *p* = 0.09,948; and Tukey's (genotype): light: WT vs SCN^Con^ *p* = 0.0272; WT vs SCN^Cry1^ *p* = 0.95 879; SCN^Con^ vs SCN^Cry1^ *p* = 0.1467; dark: WT vs SCN^Con^ *p* = 0.0232; WT vs SCN^Cry1^ *p* = 0.1612; SCN^Con^ vs SCN^Cry1^ *p* = 0.54; NREMS: interaction: *F*_(2,16)_ = 19.42, *p* < 0.0001; genotype: *F*_(2,16)_ = 1.756, *p* = 0.2065; time: *F*_(1,16)_ = 80.45, *p* < 0.0001; *post hoc* multiple comparisons tests: Sidak's (LD): WT *p* < 0.0001; SCN^Con^ *p* = 0.9967; SCN^Cry1^ *p* < 0.0001; Tukey's (genotype): light: WT vs SCN^Con^ *p* = 0.294; WT vs SCN^Cry1^ *p* = 0.5757; SCN^Con^ vs SCN^Cry1^ *p* = 0.0279; dark: WT vs SCN^Con^ *p* = 0.0056; WT vs SCN^Cry1^ *p* = 0.058; SCN^Con^ vs SCN^Cry1^ *p* = 0.4883; REMS: interaction: *F*_(2,16)_ = 4.184, *p* = 0.036; genotype: *F*_(2,16)_ = 2.032, *p* = 0.1656; time: *F*_(1,16)_ = 42.26, *p* < 0.0001; *post hoc* multiple comparisons tests: Sidak's (LD): WT *p* = 0.0006; SCN^Con^ *p* = 0.4367; SCN^Cry1^ *p* = 0.0005; Tukey's (genotype): light: WT vs SCN^Con^ *p* = 0.9696; WT vs SCN^Cry1^ *p* = 0.7682; SCN^Con^ vs SCN^Cry1^ *p* = 0.8841; dark: WT vs SCN^Con^ *p* = 0.0103; WT vs SCN^Cry1^ *p* = 0.4043; SCN^Con^ vs SCN^Cry1^ *p* = 0.119; 2×RMANOVA *post hoc* Sidak's multiple comparisons test (LD difference within genotype) ****p < 0.0001, ***p < 0.001, **p < 0.01; *post hoc* Tukey's multiple comparisons test (genotype difference) ****p < 0.0001 ***p < 0.001, **p < 0.01, *p < 0.05]. ***E***, Mean (+ SEM) number of transitions between sleep-wake states in LD reveals an increase in the numbers of transitions between wake-NREMS-wake in the SCN^Con^ mice suggesting a lack of consolidated sleep/wake (1×ANOVA: LD: wake-NREMS: *F*_(2,16)_ = 5.8, *p* = 0.012; Tukey's multiple comparisons test WT vs SCN^Con^ *p* = 0.033; WT vs SCN^Cry1^ *p* = 0.9996; SCN^Con^ vs SCN^Cry1^ *p* = 0.021: NREMS-wake: *F*_(2,16)_ = 5.5, *p* = 0.0148; Tukey's multiple comparisons test WT vs SCN^Con^ *p* = 0.0203; WT vs SCN^Cry1^ *p* = 0.788; SCN^Con^ vs SCN^Cry1^ *p* = 0.0481). ***F***, Mean (+ SEM) number of transitions between sleep-wake states in DD shows a significant increase in NREMS-wake transitions in the SCN^Con^ mice compared with both the WT and SCN^Cry1^ mice. Therefore, restoration of rhythmicity to the region of the SCN in SCN^Cry1^ mice restores sleep/wake consolidation (1×ANOVA: DD: wake-NREMS: *F*_(2,15)_ = 1.1, *p* = 0.3; NREMS-wake: *F*_(2,15)_ = 10.8 *p* = 0.0012; Tukey's multiple comparisons test WT vs SCN^Con^ *p* = 0.0044; WT vs SCN^Cry1^ *p* = 0.9995; SCN^Con^ vs SCN^Cry1^ *p* = 0.0021); *p < 0.05, **p < 0.01.

Loss of circadian patterning to sleep-wake in SCN^Con^ mice and its restoration to WT-like organization in SCN^Cry1^ mice were indicators of the autonomous power of the suprachiasmatic clock. We then examined its effect on sleep/wake architecture, as there was no a priori reason to expect that a functional SCN could reinstate a WT-like structure. When entrained to an LD cycle, WT mice showed a longer duration of wake in the dark (active) phase, and correspondingly fewer episodes of NREMs and REMS at night and more in the light (rest) phase (with no systematic changes in their duration; Fig. 6*A*,*B*; as assessed by *post hoc* Sidak's and Tukey's multiple comparisons tests within genotype and between genotypes, respectively, where there was a significant interaction and/or genotype effect in a 2×RMANOVA; see legend to Fig. 6 for details). In comparison, SCN^Con^ mice showed weaker consolidation. They did not exhibit longer wake bouts during the dark (active) phase than during the light (rest) phase in LD, with nocturnal wake bouts being shorter than in WT mice, and NREMS bouts longer in both the light and dark phases, as reported previously for *Cry1*, *Cry2*-null mice (Wisor et al., 2002). Nevertheless, in SCN^Con^ mice, although bouts of NREMS and REMS were more frequent in the light compared with the dark phase, they had significantly fewer bouts during the daytime (rest phase) than WT mice (Fig. 6*B*; as assessed by *post hoc* Tukey's multiple comparisons between genotypes where there was a significant interaction and/or genotype effect in a 2×RMANOVA; see legend to Fig. 6 for details). Local expression of Cry1 in the SCN region reversed the deficits of SCN^Con^ mice in LD: the duration of wake bouts was significantly longer at night compared with SCN^Con^ mice, and no different from WT measures, and the duration and number of NREMS and REMS bouts was comparable to WT mice (Fig. 6*A*,*B*; as assessed by *post hoc* Tukey's multiple comparisons between genotypes where there was a significant interaction and/or genotype effect in a 2×RMANOVA; see legend to Fig. 6 for details).

The differences between groups in sleep consolidation were even more stark under circadian free-running conditions. WT mice retained their longer duration of nocturnal wake bouts (active phase) and more bouts of NREMS and REMS in circadian daytime (rest phase; Fig. 6*C*,*D*; 2×RMANOVA with *post hoc* Sidak's multiple comparisons tests within genotype; see legend to Fig. 6 for statistical analyses). SCN^Con^ mice, however, exhibited no significant rest/active differences in the duration or number for any vigilance state. The durations of wake bouts in circadian night were significantly shorter than in WT mice, and the number of nocturnal bouts of all three states were more numerous than in WT mice, reflecting the loss of consolidated wake in circadian night. Equally, SCN^Con^ exhibited significantly more episodes of wake in circadian daytime than did WT mice (as assessed by *post hoc* Sidak's and Tukey's multiple comparisons tests within genotype and between genotypes, respectively, where there was a significant interaction and/or genotype effect in a 2×RMANOVA; see legend to Fig. 6 for details). Importantly, all of these deficiencies in SCN^Con^ mice were reversed by expression of Cry1 in the SCN and adjacent tissue. In SCN^Cry1^ mice, nocturnal wake bout duration was significantly longer than in subjective day, albeit not as long as in WT mice, and bouts of NREMS and REMS were significantly more numerous in circadian day than in circadian night (as assessed by *post hoc* Sidak's and Tukey's multiple comparisons tests within genotype and between genotypes, respectively, where there was a significant interaction and/or genotype effect in a 2×RMANOVA; see legend to Fig. 6 for details). Thus, global loss of Cry proteins destabilizes sleep-wake structure on LD and even more so under DD, while restoring local expression of Cry1 in the SCN region, resulting in a molecularly competent SCN, reverses the loss of sleep-wake architecture and consolidates vigilance states in SCN^Cry1^ mice.

These differences in sleep-wake consolidation between groups were emphasized further by the number of transitions between wake-NREMS and NREMS-wake (there were no significant differences in transitions between NREMS-REMS, REMS-NREMS or REMS-wake following a 1×ANOVA in either LD or DD). In both LD and DD, SCN^Con^ mice showed more transitions than did WT mice, and this was reversed in SCN^Cry1^ mice in both LD (Fig. 6*E*) and DD (Fig. 6*F*) compared with WT and SCN^Cry1^ mice (as assessed by *post hoc* Tukey's multiple comparisons tests within genotype following a 1×ANOVA; see legend to Fig. 6 for details). This further confirms that rhythmic expression of Cry1 targeted to the SCN of *Cry1/Cry2*-null mice stabilizes the sleep-wake over both the 24-h LD cycle and across CT, consistent with the view that the suprachiasmatic pacemaker drives the maintenance of wakefulness and the consolidation of sleep, as appropriate, across the LD cycle and across subjective day and night.

### Temporal control of sleep homeostasis in SCN^Cry1^ mice

**Figure 7.**
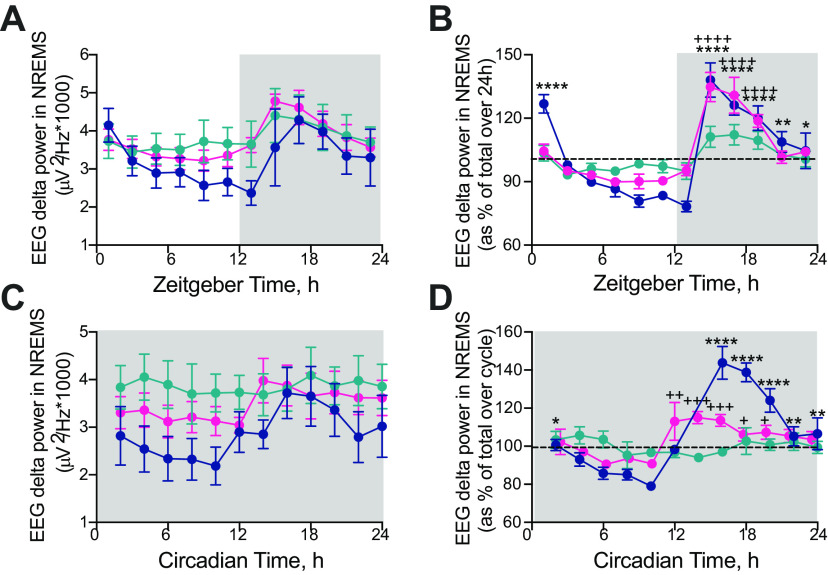
Characterization of the effect of local Cry1 expression in the suprachiasmatic hypothalamus on δ power (1–4 Hz) in NREMS in entrained and free-running conditions. ***A***, 2-h profiles (mean ± SEM) of NREMS EEG raw δ power in WT, SCN^Con^, and SCN^Cry1^ mice in entrained (LD) conditions. ***B***, 2-h profiles (mean ± SEM) of NREMS EEG relative δ power (relative to total power) in WT, SCN^Con^, and SCN^Cry1^ mice in entrained (LD) conditions reveals both the WT and SCN^Cry1^ mice have significant, appropriately phased rhythms in NREMS EEG relative power, whereas the SCN^Con^ mice have a low-amplitude rhythm (WT: 1×ANOVA: *F*_(11,47)_ = 15.8, *p* < 0.0001; *post hoc* Dunnett's multiple comparisons test vs ZT10: *p* < 0.0001 ZT2, *p* = 0.1405 ZT4, *p* = 0.7954 ZT6, *p* = 0.9825 ZT8, *p* = 0.9995 ZT12, *p* = 0.9995 ZT14, *p* < 0.0001 ZT16, *p* < 0.0001 ZT18, *p* < 0.0001 ZT20, *p* = 0.0024 ZT22, *p* = 0.0136 ZT24; SCN^Con^: *F*_(11,68)_ = 3.9, *p* = 0.0165; *post hoc* Dunnett's multiple comparisons test versus ZT10: all not significant; SCN^Cry1^: *F*_(11,70)_ = 14.5, *p* < 0.0001, *post hoc* Dunnett's multiple comparisons test versus ZT10: *p* = 0.1387 ZT2, *p* = 0.9811 ZT4, *p* = 0.9993 ZT6, *p* > 0.9999 ZT8, *p* > 0.9999 ZT12, *p* = 0.9815 ZT14, *p* < 0.0001 ZT16, *p* < 0.0001 ZT18, *p* < 0.0001 ZT20, *p* = 0.3489 ZT22, *p* = 0.1845 ZT24). ***C***, 2-h profiles (mean ± SEM) of NREMS EEG raw δ power in WT, SCN^Con^, and SCN^Cry1^ mice in constant (DD) conditions. ***D***, 2-h profiles (mean ± SEM) of NREMS EEG relative δ power (relative to total power) in WT, SCN^Con^, and SCN^Cry1^ mice in constant (DD) conditions; 1×ANOVA shows both WT and SCN^Cry1^ mice have significant circadian rhythms, but there was no significant rhythm in control-treated SCN^Con^ mice (WT: *F*_(11,40)_ = 20.1, *p* < 0.0001; *post hoc* Dunnett's multiple comparisons test versus CT10: *p* = 0.019 CT2, *p* = 0.2632 CT4, *p* = 0.9361 CT6, *p* = 0.971 CT8, *p* < 0.0001 CT14–CT20, *p* = 0.0028 CT22, *p* = 0.0032 CT24); SCN^Con^: *F*_(11,58)_ = 0.78, *p* = 0.66; SCN^Cry1^: *F*_(11,68)_ = 4.17, *p* < 0.0001, *post hoc* Dunnett's multiple comparisons test vs CT10: *p* = 0.2344 CT2, *p* = 0.859 CT4, *p* > 0.9999 CT6, *p* = 0.9992 CT8, *p* = 0.005 CT12, *p* = 0.0005 CT14, *p* = 0.0007 CT16, *p* = 0.046 CT18, *p* = 0.026 CT20, *p* = 0.0653 CT22, *p* = 0.1459 CT24). WT versus ZT/CT10: *p < 0.05, **p < 0.01, ***p < 0.001, ****p < 0.0001; SCN^Cry1^ vs ZT10/CT10: +*p* < 0.05, ++*p* < 0.01, +++*p* < 0.001, ++++*p* < 0.0001. Gray shaded area represents darkness; WT (*n* = 5), blue; SCN^Con^ (*n* = 7), green; SCN^Cry1^ (*n* = 7), magenta.

To what extent can SCN-mediated consolidation of sleep timing and patterning affect homeostasis? EEG δ power (1–4 Hz) during NREMS is a commonly used index of sleep homeostasis, with higher levels indicating increased sleep need. Under LD, absolute and relative EEG δ power in NREMS (normalized to total power to correct for electrode placement) in WT mice declined spontaneously across the inactive light phase and increased during the dark phase (Fig. 7*A*,*B*), coincident with increased wake. This pattern was clearly under circadian regulation in DD (Fig. 7*C*,*D*). In contrast, SCN^Con^ mice revealed only a low-amplitude pattern on LD, and no significant circadian pattern in DD (Fig. 7*C*,*D*). As reported previously (Wisor et al., 2002); however, *Cry1/Cry2*-null mice exhibited higher levels of absolute NREMS EEG δ power, in particular during the light/inactive phase when compared with WT mice (Fig. 7*A*,*C*). SCN^Cry1^ mice had a significant rhythm in relative δ power not only in LD but also in DD (Fig. 7*B*,*D*; as assessed by *post hoc* Dunnett's multiple comparisons tests against the time point before transition to lights off (LD)/activity onset (DD) following a 1×ANOVA; see legend to Fig. 7 for details). In LD, the peak amplitude of EEG δ power of SCN^Cry1^ mice was the same as in WT, and although in DD the peak amplitude was reduced compared with WT, it was nevertheless phased appropriately (Fig. 7*D*). This lower peak may reflect the slightly higher levels of NREMS in the early circadian active phase in DD compared with WT, a difference which was not evident in LD (Fig. 5*B*,*F*). Finally, all groups showed a similar latency to first NREMS episode >100 s on the dark-to-light transition (latency to first NREMS episode >100 s: WT = 18.9 ± 8.9 min, *n* = 5, SCN^Con^ = 18.0 ± 6.8 min, *n* = 7; SCN^Cry1^ = 17.5 ± 5.2 min, *n* = 7; 1×ANOVA: *F*_(2,16)_ = 0.01, *p* = 0.99). Together, these data demonstrate that rescuing Cry1 in the SCN region restores rhythmic expression of NREMS EEG δ power in both LD and DD, confirming the appropriate phasing and organization of sleep-wake across the 24-h/circadian cycle. SCN-mediated circadian organization and consolidation were therefore accompanied by appropriate dynamic signaling of sleep need.

**Figure 8.**
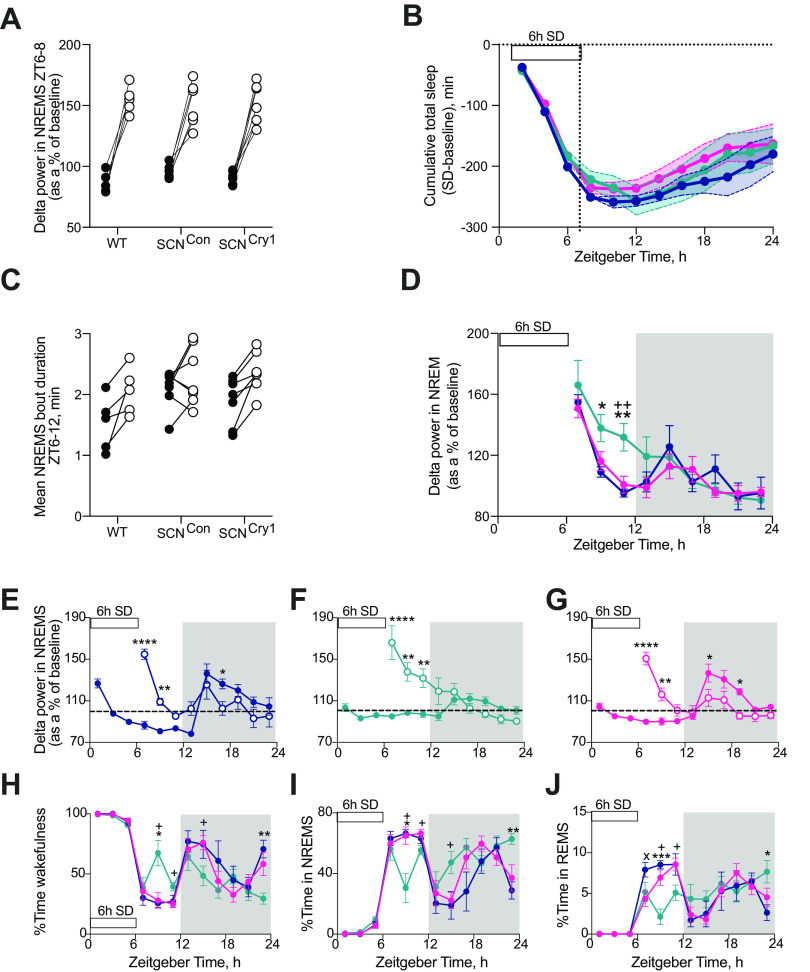
Effect of rescue of rhythmic Cry1 expression in the suprachiasmatic hypothalamus on the homeostatic response to 6-h SD. ***A***, Individual changes in NREMS δ power (1–4 Hz) in the first 2 h of recovery sleep (ZT6–ZT8; open circles) compared with baseline sleep (ZT6–ZT8; closed circles) in WT, SCN^Con^, and SCN^Cry1^mice shows a significant effect of the treatment (6-h SD) with all three genotypes showing the SD was effective in initiating a significant homeostatic response, but no genotype or genotype × treatment effects (2×RMANOVA: interaction: *F*_(2,16)_ = 0.19, *p* = 0.83; genotype: *F*_(2,16)_ = 1.19, *p* = 0.33; treatment (±6-h SD): *F*_(1,16)_ = 83.91, *p* < 0.0001). ***B***, No significant differences between genotypes in the cumulative decrease in TS time (Mean±SEM; NREMS + REMS) over the 6 h of SD with subsequent partial recovery of sleep during the 18 h post-SD in WT (*n* = 5, blue), SCN^Con^ (*n* = 7, green), and SCN^Cry1^ (*n* = 7, magenta) mice (2×RMANOVA: interaction: *F*_(22,176)_ = 0.39, *p* = 0.99; genotype: *F*_(2,16)_ = 0.43, *p* = 0.65; time: *F*_(2,24)_ = 58.5, *p* < 0.0001). ***C***, Individual changes in NREMS bout duration (minutes) between ZT6 and ZT12 on baseline day (closed circles) and after 6-h SD (open circles); although there was a significant effect of the treatment (6-h SD) in increasing the duration of the NREMS bout duration there was no genotype or genotype × treatment effect (2×RMANOVA: interaction: *F*_(2,16)_ = 1.3, *p* = 0.29; genotype: *F*_(2,16)_ = 2.25, *p* = 0.14; treatment (±6-h SD): *F*_(1,16)_ = 20.15, *p* = 0.0004). ***D***, Comparison of the time course of EEG δ power (Mean±SEM) in NREMS following 6 h of SD between WT (blue), SCN^Con^ (green), and SCN^Cry1^ (magenta) mice. The decline in EEG δ power in NREMS was significantly slower in SCN^Con^ mice when compared with WT and SCN^Cry1^ animals (2×RMANOVA: interaction: *F*_(16,123)_ = 2.33, *p* = 0.0049, genotype: *F*_(2,16)_ = 0.91, *p* = 0.4235; time: *F*_(4,55)_ = 27.22, *p* < 0.0001; *post hoc* Tukey's multiple comparison test *p < 0.05, **p < 0.01 WT vs SCN^Con^; ++*p* < 0.01 SCN^Con^ vs SCN^Cry1^). ***E–G***, Time course (mean ± SEM) of EEG δ power relative to baseline during NREMS on baseline day (closed circles) and following 6-h SD (open circles; data replotted from ***D***) in WT (***E***; *n* = 5, blue), SCN^Con^ (***F***; *n* = 7, green), and SCN^Cry1^ (***G***; *n* = 7, magenta) mice [2×RMANOVA: WT: interaction: *F*_(8,27)_ = 14.63, *p* < 0.0001; time: *F*_(8,32)_ = 6.43, *p* < 0.0001, treatment (+6-h SD) *F*_(1,4)_ = 6.34, *p* < 0.0001; SCN^Con^: interaction: *F*_(8,44)_ = 13.54, *p* < 0.0001; time: *F*_(8,48)_ = 7.54, *p* < 0.0001, treatment (+6-h SD) *F*_(1,6)_ = 6.0, *p* = 0.0498; SCN^Cry1^: interaction: *F*_(8,45)_ = 17.84, *p* < 0.0001; time: *F*_(8,48)_ = 12.14, *p* < 0.0001, treatment (+6-h SD) *F*_(1,6)_ = 60.28, *p* = 0.613; *post hoc* Sidak's multiple comparison test baseline vs +6-h SD *p < 0.05, **p < 0.01, ***p < 0.001, ****p < 0.0001]. ***H–J***, Percentage time (mean ± SEM) spent in wakefulness, NREMS and REMS during 6 h of SD and 18 h of recovery in WT (*n* = 5, blue), SCN^Con^ (*n* = 7, green), and SCN^Cry1^ (*n* = 7, magenta) mice. The SCN^Con^ mice show significant increases in wake with concomitant decreases in NREMS and REMS during the recovery from 6-h SD, in particular during the light phase (ZT6–ZT12), and at the end of the dark phase (2×RMANOVA: wake: interaction: *F*_(22,176)_ = 3.05, *p* < 0.0001, genotype: *F*_(2,16)_ = 0.76, *p* = 0.49; time: *F*_(6,89)_ = 40.96, *p* < 0.0001; NREMS: interaction: *F*_(22,176)_ = 3.43, *p* < 0.0001, genotype: *F*_(2,16)_ = 1.54, *p* = 0.2436; time: *F*_(6,97)_ = 46.45, *p* < 0.0001; REMS: interaction: *F*_(22,176)_ = 2.97, *p* < 0.0001, genotype: *F*_(2,16)_ = 0.11, *p* = 0.8982; time: *F*_(6,93)_ = 24.01, *p* < 0.0001; *post hoc* Tukey's multiple comparison test *p < 0.05, **p < 0.01, ***p < 0.001 WT vs SCN^Con^; x*p* < 0.05 WT vs SCN^Cry1^; +*p* < 0.05 SCN^Con^ vs SCN^Cry1^).

Having shown that the suprachiasmatic clock can direct the circadian patterning and stabilization of sleep-wake cycles, we next tested whether restoring a molecularly competent clock in the SCN region has an effect on the homeostatic regulation of sleep by measuring sleep and the EEG responses following 6-h SD that started from lights onset (ZT0–ZT6). SD was equally effective across the three groups, with no significant differences in the small amount of NREMS during SD (WT 9.8 ± 3.5 min, SCN^Con^ 13.6 ± 2.7 min, SCN^Cry1^ 7.4 ± 2.5 min, *n* = 5, 7, 7, respectively; 1×ANOVA: *F*_(2,16)_ = 1.61, *p* = 0.2311) or the latency to sleep after SD (time to first NREMS bout >100s duration, WT 12.9 ± 6.8 min; SCN^Con^ 10.4 ± 3.4 min; SCN^Cry1^ 10.3 ± 3.7 min; 1×ANOVA: *F*_(2,16)_ = 0.18, *p* = 0.8354). In the 2 h immediately after SD, all groups showed a significant increase in δ power when NREMS occurred in that interval reflecting greater homeostatic sleep pressure (Fig. 8*A*; no significant interaction or genotype effect following 2×RMANOVA showing that all genotypes responded to the 6-h SD in the same way; see legend to Fig. 8 for statistical analyses). Genotype did not, therefore, affect the neurophysiological capacity to sense and respond to SD during subsequent NREMS when it did occur (Wisor et al., 2002). Furthermore, recovery from SD, compared with baseline, was not different between genotypes, insofar as accumulated sleep loss increased during SD, but then decreased at the same rate in all three groups over the subsequent 18 h (Fig. 8*B*). In addition, over the 6 h immediately following SD (ZT6–ZT12), the genotype of the mice did not significantly affect the change in NREMS bout duration compared with baseline, although the individual WT and SCN^Cry1^ mice all showed an increase following 6-h SD, the response was more variable in the SCN^Con^ animals (Fig. 8*C*; no significant interaction or genotype effect following 2×RMANOVA; see legend to Fig. 8 for statistical analyses; Fig. 8*C*). By whatever mechanism, all three groups recovered lost sleep, i.e., exhibited effective homeostasis.

Notwithstanding overall comparability between genotypes, there were also informative differences. The time course for the decline in EEG δ power in NREMS after SD was different. It declined progressively across the light phase in WT mice, but more slowly in SCN^Con^ mice and, unlike in WT mice, did not reach baseline levels during the light phase (Fig. 8*D–F*). Expression of Cry1 in the SCN region corrected these deficits (Fig. 8*C*,*D*,*G*). Thus, although there were no significant differences in the overall recovery of sleep loss after SD (Fig. 8*B*), there were significant differences in its time course during the light phase (ZT6–ZT12; Fig. 8*H–J*). Whereas WT and SCN^Cry1^ mice showed a sustained absence of wake (Fig. 8*H*) and elevation of both NREMS and REMS in the light phase (Fig. 8*I*,*J*), the SCN^Con^ mice exhibited significantly less (∼70 min) NREMS and more (∼60 min) wakefulness between ZT6–ZT12 following 6-h SD (1×ANOVA: wake: *F*_(2,16)_ = 6.72, *p* = 0.0076, Tukey's *post hoc* multiple comparisons tests: WT vs SCN^Con^ *p* = 0.017, WT vs SCN^Cry1^ *p* = 0.9527, SCN^Con^ vs SCN^Cry1^ *p* = 0.017; NREMS: *F*_(2,16)_ = 5.76, *p* = 0.013, Tukey's *post hoc* multiple comparisons tests: WT vs SCN^Con^ *p* = 0.034, WT vs SCN^Cry1^ *p* = 0.9995, SCN^Con^ vs SCN^Cry1^ *p* = 0.022). This suggests that SCN^Con^ mice had a decreased sleep pressure and/or an inability to maintain consolidated NREMS at this phase of the LD cycle. Given that analysis of NREMS EEG δ power indicated that decreased sleep pressure was not the case in SCN^Con^ mice (Fig. 8*A*,*D*,*F*), consolidation of NREMS bout duration may have been limiting, as all WT and SCN^Cry1^ mice increased the NREMS bout duration following 6-h SD (Fig. 8*C*), overall the SCN^Con^ mice did not show a significant difference in this parameter ±6-h SD (two-tailed paired *t* test *t*_(6)_ = 1.15, *p* = 0.2925; Fig. 8*C*). It may be that poor consolidation and/or less time in NREMS at this phase (ZT6–ZT12) altered the time course to recovering sleep loss, and may reflect an interaction between the circadian and homeostatic processes regulating sleep-wake at the end of the light phase (Fig. 8*D*,*F*,*I*). Loss of Cry proteins did not, therefore, globally affect neurophysiological mechanisms of homeostatic sleep recovery, but in SCN^Con^ mice with an ineffective SCN clock, the dynamics of recovery were altered and the expression of Cry1 in the SCN region corrected this (Fig. 8*C–J*). Cry proteins and a competent SCN clock are not, therefore, necessary components of the fundamental sleep homeostatic mechanism, but they do regulate its time course.

### Rescue of sleep-dependent memory in the novel object test in SCN^Cry1^ mice

**Figure 9.**
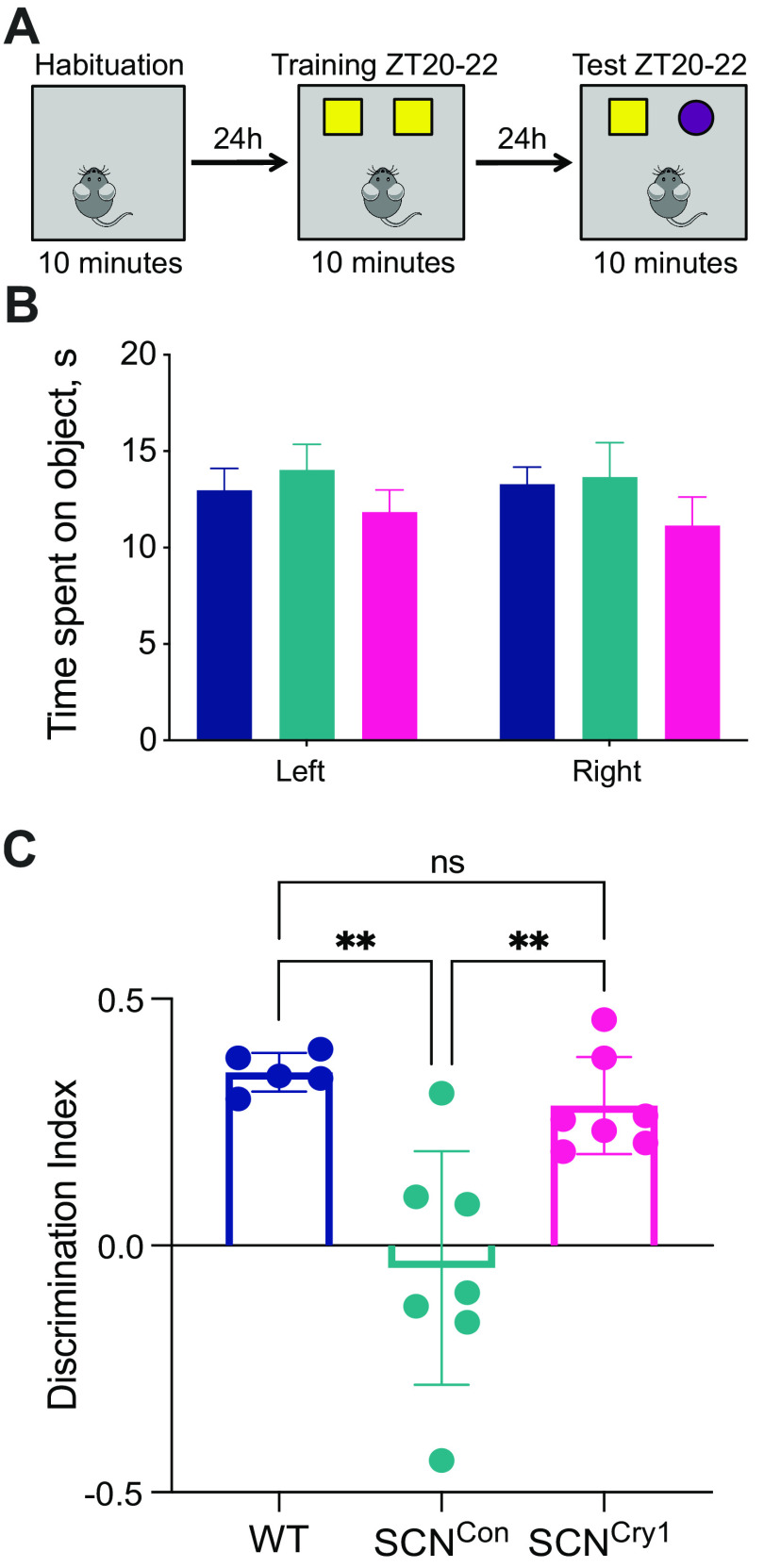
Cry1 expression in the suprachiasmatic hypothalamus rescues performance in the novel object test. ***A***, Protocol for the sleep-dependent memory test where mice are habituated to the test arena; 24 h later, mice investigate two identical objects, then 24 h later, one object is replaced with a novel object. Training and testing were done during the dark/active phase between ZT20 and ZT22. ***B***, The amount of time (mean ± SEM) the mice spent on the objects during the training phase was not significantly different between groups (see Results). ***C***, The DI (mean ± SEM and individual points) revealed the SCN^Con^ mice had an overall null preference for the novel object, whereas both the WT and SCN^Cry1^ groups of mice showed a significant preference for the novel object (see Results; 1×ANOVA with *post hoc* Tukey's multiple comparison test: WT vs SCN^Con^ **p < 0.01, WT vs SCN^Cry1^ n.s., SCN^Con^ vs SCN^Cry1^ **p < 0.01); WT (*n* = 5), blue; SCN^Con^ (*n* = 7), green; SCN^Cry1^ (*n* = 7), magenta.

To determine whether SCN-mediated circadian control over phasing and consolidation of sleep/wake cycles has consequences for brain function, we assessed cognitive performance in the NOR task, a recognized sleep-dependent behavior (Palchykova et al., 2006). There were no statistically significant differences between groups in time spent exploring the objects during training (Fig. 9*A*,*B*; 2×RMANOVA: interaction: *F*_(2,16)_ = 0.4099, *p* = 0.6705; object: *F*_(1,16)_ = 0.1558, *p* = 0.6982; group: *F*_(2,16)_ = 0.8779, *p* = 0.4348). WT mice demonstrated robust memory for the familiar object by spending significantly more time investigating the novel object when tested (positive DI). In contrast, SCN^Con^ mice failed to discriminate between the novel and familiar objects, with an overall null preference between the two objects (Fig. 9*C*). Thus, the global absence of Cry proteins compromised performance in a test of memory known to be sleep-dependent. The initiation of circadian competence in SCN^Cry1^ mice, and thereby organization of sleep/wake cycles, resulted in all seven SCN^Cry1^ mice showing a preference for the novel object (1×ANOVA *F*_(2,16)_ = 11.58, *p* = 0.0008; *post hoc* Tukey's multiple comparison test: WT vs SCN^Con^ *p* = 0.0015, WT vs SCN^Cry1^ *p* = 0.7493, SCN^Con^ vs SCN^Cry1^ *p* = 0.0035). These results demonstrate that global loss of Cry1 proteins compromises NOR performance, and that a molecularly competent suprachiasmatic pacemaker cannot only establish the necessary organization and consolidation of sleep/wake, but also sustain sleep-dependent memory.

## Discussion

To address the specific role of the SCN in sleep regulation we used genetically clockless *Cry1/Cry2*-null mice that in the absence of LD have no circadian patterning to sleep-wake cycles, poorly consolidated sleep and wake, compromised dynamics of homeostatic recovery sleep, and impaired sleep-dependent memory (van der Horst et al., 1999; Wisor et al., 2002, 2008; Maywood et al., 2018). We initiated *de novo* circadian rhythmicity locally to the SCN and closely adjacent hypothalamus by virally mediated expression of Cry1 (Edwards et al., 2016; Maywood et al., 2018). Importantly, the SCN was the only tissue successfully targeted in all mice, but for caution we refer to the SCN region. The rest of the brain, and the periphery remained circadian incompetent. In agreement with previous studies we established circadian locomotor activity rhythms in SCN^Cry1^ mice (Maywood et al., 2018) comparable to the effect of WT SCN grafts in *Cry1/Cry2*-null mice (Sujino et al., 2003). This allowed us to test the contribution of the suprachiasmatic molecular clockwork to the temporal regulation of sleep. Genetic rescue established sleep-wake cycles that were appropriately phased and consolidated, and accompanied by improved performance in a test of sleep-dependent memory. On some measures SCN^Cry1^ mice did not reach those of WTs, which may reflect the incomplete targeting of the paired SCN. Nevertheless, the data suggest that the circadian system promotes wake in subjective night and facilitates sleep, by promoting its intensity and consolidation, in subjective daytime. Although the neural circuits and neurophysiological processes underlying sleep homeostasis are not compromised by global Cry1,2 deficiency, our data do illustrate an interaction between the homeostatic and circadian mechanisms during the recovery from SD. We conclude that the SCN has continuous influence on sleep-wake organization across the circadian cycle and, directly or indirectly, modulates sleep consolidation and homeostatic regulation. Further dissection of the relevant neural pathways would require Cre-dependent targeting of specific subpopulations, such as VIP and VIP- or dopamine-receptor-expressing cells (Patton et al., 2020; Hamnett et al., 2021; Maywood et al., 2021) within the SCN and/or its targets. Moreover, analysis of female mice, in which the SCN direct estrous variability in arousal state, may provide additional insight.

Overall sleep-wake distributions across the entrained and free-running cycles showed modest differences between groups, with *Cry1,Cry2*-null mice (SCN^Con^ and SCN^Cry1^) showing an overall increase in the amount of NREMS in LD (Wisor et al., 2002). Studies using other global circadian mutations/deletions or ablation of the SCN in otherwise intact animals (Naylor et al., 2000; Easton et al., 2004; Laposky et al., 2005; Mistlberger, 2005) have demonstrated either no differences or an increase/decrease in NREMS, making it difficult to interpret whether any phenotypes are because of an extra-SCN circadian effect, or a more global effect on the dynamics of the complex neural circuitry underlining the control of sleep-wake states (Saper et al., 2010). Nevertheless, the SCN^Con^, but not the SCN^Cry1^ mice, did show a significant decrease in the amount of NREMS in the second half of the light/rest phase, suggesting that restoring rhythmicity to the SCN enables promotion of sleep/inhibition of wake at a time when the homeostatic pressure to sleep has declined. Furthermore, whereas the rescued animals showed a WT-like consolidation of wake episodes in the dark/active phase in both LD and DD, the SCN^Con^ mice did not, and this was reflected in their reduced amplitude or absence of rhythmic time course for NREMS δ power in LD and DD, respectively. Together, these results show the mouse SCN clock has opposing influences on sleep-wake organization across the cycle: promoting wakefulness in the dark/subjective day and sleep in light/subjective night.

The dynamics of sleep homeostasis were examined following SD, to assess whether there is an interaction between circadian and homeostatic regulatory processes. Evidence suggests influences of sleep homeostasis on the functioning of the circadian clock (Deboer et al., 2003, 2007; Schmidt et al., 2009), and that these processes can act independently (Tobler et al., 1983; Shiromani et al., 2004). Conversely, global circadian clock mutants can also show altered changes in NREMS EEG δ power in response to SD, consistent with a role for clock genes in sleep homeostasis (Naylor et al., 2000; Wisor et al., 2002; Laposky et al., 2005; Dijk and Archer, 2010; Curie et al., 2013). As discussed above, however, this does not necessarily indicate a role for the SCN in regulating homeostasis. Indeed, *Cry1,Cry2*-null mice, and mice lacking the Cry2 gene (SCN^Cry1^) have an intact sleep homeostatic response (Wisor et al., 2002, 2008), suggesting that the SCN is not necessary for the expression of the initial neurophysiological response to SD. Nevertheless, SCN^Con^ mice had an altered time course in their recovery from SD, most notably between ZT6 and ZT12 when the SCN is exerting a sleep-promoting influence. This suggests a role, direct or indirect, for the circadian timing system in modulating the recovery from SD, and implies an interaction between the homeostatic and circadian processes, to ensure prolonged and consolidated sleep at a phase when sleep pressure is low. Studies in humans have similarly postulated a role for the circadian system in influencing sleep homeostasis (Lazar et al., 2015), although it remains to be established whether the central circadian clock in the SCN, and/or clocks in other brain areas underlie these mechanisms.

How might the SCN exert its effects? The sleep-wake regulatory circuit has been described as a “flip-flop” switch, whereby sleep-wake transitions are regulated by a reciprocal inhibition between sleep-promoting and wake-promoting nodes of the hypothalamus and brainstem (Saper et al., 2010). Multiple direct and indirect pathways from the SCN to both sleep-promoting or wake-promoting nodes could therefore influence state-switching over the circadian cycle, which would be expected if the circadian clock has an ongoing active role in regulating sleep-wake (Deurveilher et al., 2002; Schwartz et al., 2011). Recent work has further confirmed that the circadian regulation of wakefulness is modulated by the SCN-PVN-lateral hypothalamic (LH) pathway (Ono et al., 2020). Activation of LH-GABA neurons can exert direct synaptic control over the sleep-promoting-galaninergic neurons in the ventrolateral preoptic nucleus (VLPO) promoting arousal during NREMS in the light phase (Venner et al., 2019). In addition, a LH-thalamic reticular nucleus (TRN)-GABAergic-thalamocortical inhibitory circuit may be involved in the rapid arousal during NREMS-wake transitions (Herrera et al., 2016). Similarly, the loss of the widely projecting orexin/hypocretin neurons results in more frequent transitions into sleep and so would prevent prolongation of wake episodes (Hara et al., 2001). It may be that changes in GABAergic/glutamatergic drive impose the rapid changes in state, whereas neuropeptides such as galanin/orexin act as neuromodulators influencing the stabilization of sleep-wake states, and low (no?) amplitude and/or phasing of output from these cells underlie the sleep phenotypes in *Cry1,Cry2*-null, which are ameliorated following rescue of rhythmicity in the SCN^Cry1^ mice (Willie et al., 2003; Herrera et al., 2016; Venner et al., 2019). In addition, the SCN may also act indirectly; initiation of behavioral rhythmicity in SCN^Cry1^ mice will in turn regulate their metabolic demands, providing feedback from the periphery and/or brain regions. These could in turn influence, indirectly, the timing and homeostatic regulation of sleep (Ehlen et al., 2017; Northeast et al., 2020). For example, overexpression of BMAL1 in skeletal muscle (but not the brain) is reported to influence the daily amount of NREMS, but not the 24-h pattern to sleep/wake, nor the homeostatic responses to SD (Ehlen et al., 2017).

Sleep-dependent memory was severely compromised in SCN^Con^ mice, consistent with other reports of cognitive impairment in *Cry1/Cry2*-nulls (Van der Zee et al., 2008; De Bundel et al., 2013). This could be a result of arrhythmia (in the SCN and/or hippocampal formation), or a non-circadian, molecular consequence of local Cry deficiency. Restoration in SCN^Cry1^ mice refuted the latter, emphasizing the central importance of circadian organization to cognitive function, be it in the SCN and/or locally in the hippocampal formation, and driven by the SCN. The role of the SCN may, however, be bivalent. In hamsters made arrhythmic using a light pulse paradigm, memory was impaired (Ruby et al., 2008), but the effect was reversed by SCN ablation (Fernandez et al., 2014), suggesting that a dysfunctional SCN signal is more cognitively debilitating than no signal at all. Similarly, in a mouse model of down syndrome, impaired object recognition is restored by SCN ablation (Chuluun et al., 2020). These observations raise the possibility that cognitive deficits might be mitigated by improving circadian amplitude when it is disrupted as, for example, in patients with Alzheimer's disease (Hatfield et al., 2004; Leng et al., 2019).

In conclusion, by adopting a gain-of-function approach, we have shown that the suprachiasmatic clockwork can impose temporal organization on the sleep-wake cycle, facilitating circadian initiation and maintenance of wake, promoting sleep consolidation, the dynamics of homeostatic recovery, and sleep-dependent memory. Thus, expression of Cry proteins outside the SCN region is not necessary to sustain these processes. Our results therefore add to understanding of the relative contributions of the SCN, extra-SCN clocks and circadian clock genes in the temporal organization of sleep and wake.
